# Measurements of growing surface tension of amorphous–amorphous interfaces on approaching the colloidal glass transition

**DOI:** 10.1038/s41467-018-02836-6

**Published:** 2018-01-26

**Authors:** Divya Ganapathi, K. Hima Nagamanasa, A. K. Sood, Rajesh Ganapathy

**Affiliations:** 1 Department of Physics, Indian Institute of Science, Bangalore, 560012 India; 2 Chemistry and Physics of Materials Unit, Jawaharlal Nehru Centre for Advanced Scientific Research, Jakkur, Bangalore, 560064 India; 3 International Centre for Materials Science, Jawaharlal Nehru Centre for Advanced Scientific Research, Jakkur, Bangalore, 560064 India; 4 Sheikh Saqr Laboratory, Jawaharlal Nehru Centre for Advanced Scientific Research, Jakkur, Bangalore, 560064 India; 5 Present Address: IBS Center for Soft and Living Matter, UNIST, Ulsan, 689-798 South Korea

## Abstract

There is mounting evidence indicating that relaxation dynamics in liquids approaching their glass transition not only become increasingly cooperative, but the relaxing regions also become more compact in shape. Of the many theories of the glass transition, only the random first-order theory—a thermodynamic framework—anticipates the surface tension of relaxing regions to play a role in deciding both their size and morphology. However, owing to the amorphous nature of the relaxing regions, even the identification of their interfaces has not been possible in experiments hitherto. Here, we devise a method to directly quantify the dynamics of amorphous–amorphous interfaces in bulk supercooled colloidal liquids. Our procedure also helped unveil a non-monotonic evolution in dynamical correlations with supercooling in bulk liquids. We measure the surface tension of the interfaces and show that it increases rapidly across the mode-coupling area fraction. Our experiments support a thermodynamic origin of the glass transition.

## Abstract (web summary)

The existence of interfaces, separating distinct relaxing regions, has been predicted in glass  theory, but a direct proof remains challenging due to the amorphous nature of glasses. Ganapathi et al. identify and measure the surface tension of these interfaces in bulk supercooled colloidal liquids.

## Introduction

The precise mechanisms by which liquids vitrify, upon rapid cooling, continue to elude our grasp^1–3^. Of the many competing theories, both thermodynamic^4–8^ and kinetic^9–11^, that attempt to capture the dynamical slowing down during glass formation, the well-developed thermodynamic framework of random first-order theory (RFOT) has recently gained prominence. RFOT anticipates two transitions en route to forming glass^1,12^. The first, a purely dynamical transition at *T*_c_ > *T*_K_ (*T*_c_ and *T*_K_ being the mode-coupling and Kauzmann temperatures, respectively) is associated with the fragmentation of the homogeneous liquid into a patchwork of distinct amorphous mosaics, separated by well-defined interfaces. The competition between the configurational entropy gain and the interfacial energy cost following a rearrangement sets the size of these mosaics, which in turn governs the structural relaxation time, *τ*_*α*_. With further supercooling, the mosaic size is expected to grow and eventually diverge at a bona fide thermodynamic transition to an ideal glass at *T*_K_^1,3^. A non-zero surface tension, *ϒ*, is essential for the stability of these mosaics^13–15^ and is perhaps the most fundamental prediction of RFOT. Nevertheless, even identifying these interfaces, let alone quantify the evolution of *ϒ* across *T*_c_, has not been possible in bulk liquids. Instead, motivated by measurements of the mosaic size, also called the point-to-set length *ξ*_PTS_, in particle pinning based methods^16^, theoretical and numerical studies have attempted to use this method to quantify *ϒ*^13,14,17^ and also probe the statistics of interface fluctuations^15,16,18^. While *ϒ* was estimated to grow monotonically across *T*_c_, the study focused on inherent structures and moreover measuring *ϒ* also involved swapping particles within a cavity keeping the boundary ones frozen and hence has no experimental analogue^13^. Simulations that probed the dynamics of a liquid near a pinned amorphous wall have also uncovered direct evidence for the predicted change in relaxation mechanism across *T*_c_^19^, in terms of a maximum in the dynamic correlation length, *ξ*_d_^20^. Subsequent colloid experiments that mimicked the simulation protocol not only corroborated these findings but also showed that the maximum in *ξ*_d_ coincides with the change in shape of most-mobile particle clusters from string like to compact^21,22^. This observation was at odds with the dynamical facilitation theory of glasses^2,11^.

Even while the artificial introduction of disorder by pinning particles seems to be a prerequisite for testing predictions from RFOT^13–16,20,21,23–26^, whether these findings readily carry over to structural glasses, where disorder is self-pinned^1^, remains unanswered. At present, even in numerical studies on bulk supercooled liquids, evidence for a change in relaxation dynamics across *T*_c_ is rather indirect^27,28^ leading to suggestions that the non-monotonicity in *ξ*_d_ may be unique to the pinned wall geometry^29–31^. These concerns notwithstanding, simulations find that a pinned wall can subtly influence particle dynamics by exerting entropic forces that depend on the nature of the inter-particle potential^32^. Similar problems persist even when the particles are randomly pinned. Although increasing the concentration of the pins results in a substantial growth in *τ*_*α*_, the peak in the dynamic susceptibility, $\chi _{\mathrm{4}}^ \ast$, related to the size of dynamical heterogeneities, remains nearly constant^33^ or is found to decrease^34^ depending on the system under consideration. This behavior of $\chi _{\mathrm{4}}^ \ast$ is unlike what is observed in bulk liquids, where it steadily grows with supercooling, and suggests that the nature of relaxation dynamics in the pinned liquid may be quite different from the bulk. Dynamics aside, there is no consensus on whether *ξ*_PTS_ is even order agnostic and tracks structural correlations that are different from those obtained from simple pair-correlations^35,36^. Developing strategies to help resolve these controversies is a much needed step towards solving the glass transition puzzle.

Using the data acquired from optical video microscopy experiments on bulk supercooled colloidal liquids (see Methods section for details), here we devise a novel scheme to identify self-induced pins and probe their influence on local structure and dynamics. We exploit this conceptual advance to side step controversies surrounding the pinning procedure and directly measure the surface tension of the interfaces delineating regions of high and low configurational overlap. Apart from a growing static length scale, we also observe a non-monotonic evolution in the dynamic length scale, with supercooling, even in the absence of externally introduced pinning. Using the capillary fluctuation method (CFM)^37–41^, we calculate the surface tension of the interfaces and show that it grows rapidly on approaching the mode-coupling area fraction as anticipated by RFOT.

## Results

### Evolution of length scales in bulk colloidal glass formers

**Fig. 1:**
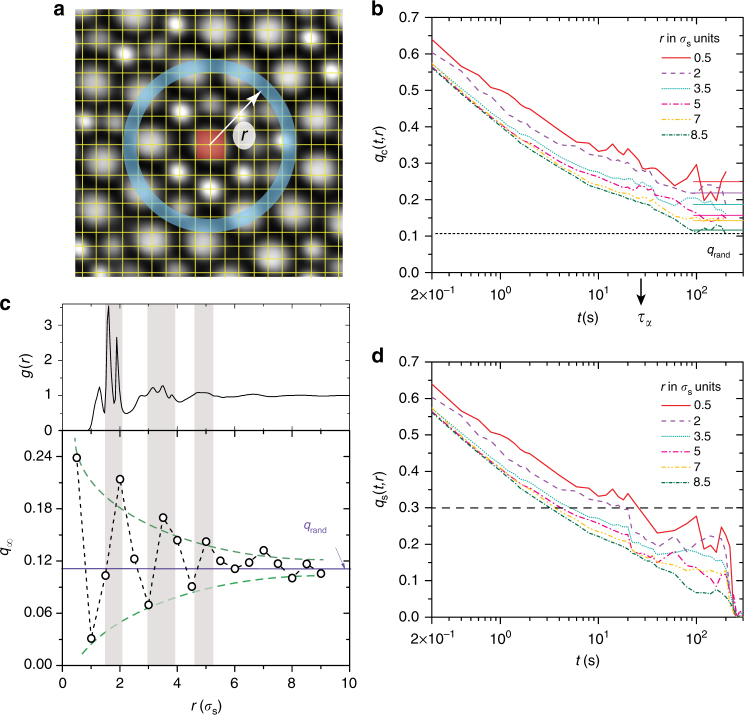
Configurational and self-overlap around self-induced pins. **a** The background image represents a small portion of the field of view of a colloidal supercooled liquid. The grid corresponds to a box size of 0.5*σ*_s_. The red box in the center of the image represents a self-induced pin with *q*_c_(*τ*_*α*_) > 0.9 over a 1*σ*_s_ box size. Upon fine-graining to 0.5*σ*_s_ boxes to improve the spatial resolution, the overlap of the self-induced pin is shared by four boxes within the red region. 〈*q*_c_(*t*)〉_*r*_ is obtained by radially averaging boxes at a distance *r* (blue ring) from the center. **b** Time evolution of configurational overlap *q*_c_(*t*,*r*) for *ϕ* = 0.74 at different *r*'s from the self-induced pin. The horizontal lines represents *q*_∞_(*r*). The horizontal red dashed line represents the random occupancy in the bulk *q*_rand_. **c** Top panel shows *g*(*r*) for the binary supercooled liquid at *ϕ* = 0.74 and the bottom panel shows the evolution of *q*_∞_ around a self-induced pin. The purple line corresponds to the bulk value *q*_rand_. The green lines are only a guide to the eye. For the sake of clarity, **b** shows *q*_c_(*t*,*r*) only for *r*'s where *q*_∞_ > *q*_rand_. **d** Time evolution of self-overlap *q*_s_(*t*,*r*) for *ϕ* = 0.74 at different distances (*σ*_s_) from the self-induced pin. The horizontal red dashed line corresponds to *q*_s_(*t*,*r*) = 0.3

We identify self-induced pins by exploiting the fact that *τ*_*α*_ is determined by the slowest relaxing regions in the supercooled liquid and hence regions that harbor these pins should also be configurationally similar over at least *τ*_*α*_. The configurational overlap *q*_c_(*t*) which measures the extent of this similarity over time is also thought to be the order parameter within RFOT^1,12^. We measure *q*_c_(*t*) by first coarse-graining the field of view into boxes of size 1*σ*_s_ (Fig. 1a). The box size is optimally chosen to minimize the overlap fluctuations due to cage rattling as well as avoid multi-particle occupancy in a given box^20,21^. Nevertheless, particles located near the box edges can still hop to neighboring boxes and this results in spurious overlap fluctuations. We account for these by developing the fuzzy-grid method which involves displacing the coarse-graining grid by the cage size 0.1*σ*_s_ in various directions and then averaging *q*_c_(*t*) obtained from each of the realizations (Supplementary Note 1 and Supplementary Fig. 1). For each box and for *t* = *τ*_*α*_, we compute $q_{\mathrm{c}}(t) = \frac{{\langle n_i(t)n_i(0)\rangle _t}}{{\langle n_i(0)\rangle _t}}$, where *i* is the box index, *n*_*i*_(*t*) = 1 if the box contains a particle at time *t* and *n*_*i*_(*t*) = 0 otherwise. Unlike the case of the quenched disorder, self-induced pins do not persist indefinitely since the liquid eventually relaxes and hence the time averaging denoted by 〈〉_*t*_ is performed over 1*τ*_*α*_. Boxes with a *q*_c_(*τ*_*α*_)>0.9 are identified as self-induced pins (red box in Fig. 1a and Supplementary Fig. 2) and we restrict our attention to those that persisted over many consecutive *τ*_*α*_'s. For *ϕ* = 0.79>*ϕ*_MCT_, however, owing to experimental difficulties with sample equilibration, *t* = 7t*, where t* is the cage-breaking time^21,42^ (Supplementary Fig. 3).

Having identified the pins, we probe their influence on local static order and dynamics by adapting the procedure originally developed for the amorphous wall geometry^20^. In order to improve the spatial resolution, the coarse-graining box size was lowered to 0.5*σ*_s_. Next, we compute the radially averaged configurational overlap *q*_c_(*t*,*r*) = 〈*q*_c_(*t*)〉_*r*_ and the self-overlap $q_{\mathrm{s}}(t,r) = \langle \frac{{\langle n_i^{\mathrm{s}}(t)n_i^{\mathrm{s}}(0)\rangle _t}}{{\langle n_i^{\mathrm{s}}(0)\rangle _t}}\rangle _r$ for all boxes at a distance *r* from a given pin. Here, $n_i^{\mathrm{s}}(t) = 1$ if the box is occupied by the same particle at time *t* and $n_i^{\mathrm{s}}(t) = 0$ otherwise. By construction, *q*_c_(*t*,*r*) is insensitive to particle swaps, while *q*_s_(*t*,*r*) is sensitive. Figure 1b shows the time evolution of *q*_c_(*t*,*r*) for *ϕ* = 0.74 at different *r*'s. The long time value of *q*_c_(*t* → ∞,*r*) = *q*_∞_ is proportional to the extent to which self-induced pins influence the local static density field. We obtain *q*_∞_ by averaging over the plateau region of *q*_c_(*t*,*r*) (horizontal lines in Fig. 1b). For large *r*'s, as expected, *q*_∞_ hovers around the bulk value *q*_rand_ = *q*_∞_(*r* → ∞) (dashed line in bottom panel of Fig. 1c). Here, *q*_rand_ measures the probability that a box is occupied. For small *r*, however, we observe oscillations in *q*_∞_ centered around *q*_rand_. By the very definition of *q*_c_(*t*,*r*), we expect *q*_∞_ for *r*'s corresponding to predominantly filled(empty) boxes to be larger(smaller) than *q*_rand_. Not surprisingly, the maxima and minima in *q*_∞_ coincide with those observed in the pair-correlation function, *g*(*r*), and indeed correspond to predominantly filled and empty coordination shells around the self-induced pin (top panel of Fig. 1c). The oscillations thus reflect the local liquid-like order around the pin and grow in amplitude on nearing the pin and indicate that density fluctuations become increasingly frozen. Since *q*_c_(*t*,*r*) is a measure of persistence of configurations, the oscillations in *q*_∞_ are more stark even at large *r* in comparison to *g*(*r*)—a purely static measure. Unlike *q*_c_(*t*,*r*), *q*_s_(*t*,*r*) decays to zero in the long time limit, when particles have undergone displacements larger than the box size (Fig. 1d). We define the relaxation time *τ*_s_(*r*) as the time taken for *q*_s_(*t*,*r*) to decay to 0.3. For all *ϕ*s, the lifetime of the self-induced pins considered is larger than *τ*_s_(*r*) and the pins therefore mimic quenched random disorder. As expected, *τ*_s_(*r*) close to the pin is larger than its bulk value $\tau _{\mathrm{s}}^{{\mathrm{bulk}}}$.

**Fig. 2:**
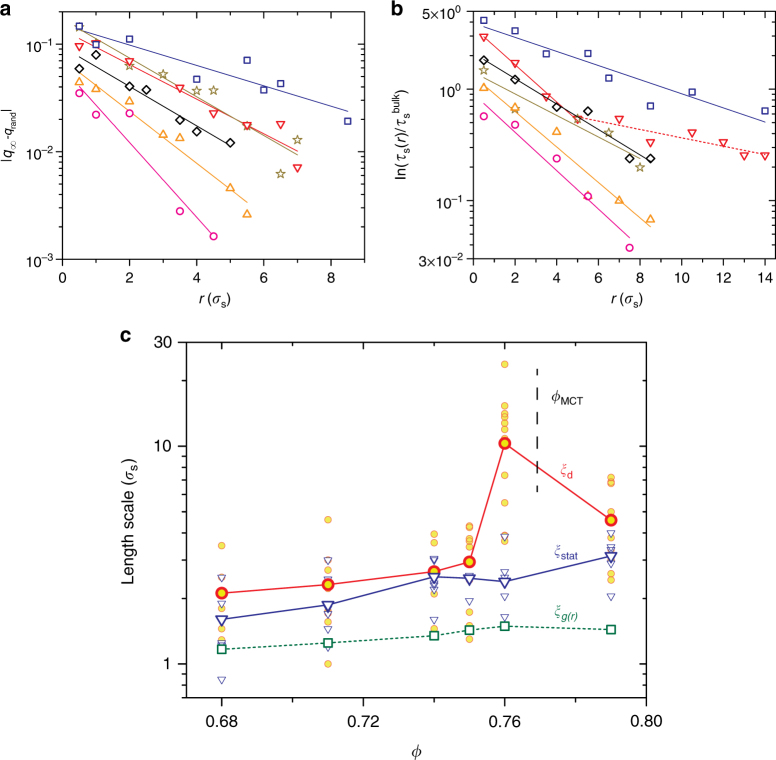
Static and non-monotonic dynamic correlations in unpinned supercooled liquids. **a** and **b** correspond to |*q*_∞_(*r*)−*q*_rand_| and ${\rm ln}(\tau _{\mathrm{s}}(r)/\tau _{\mathrm{s}}^{{\mathrm{bulk}}})$ versus *r*, respectively. *ϕ* = 0.68 (pink circles), *ϕ* = 0.71 (orange triangles), *ϕ* = 0.74 (gray diamonds), *ϕ* = 0.75 (dark yellow stars), *ϕ* = 0.76 (red inverted triangles), and *ϕ* = 0.79 (blue squares). **c** The small circles and triangles represent *ξ*_d_ and *ξ*_stat_, respectively, for many independent pins for a given *ϕ* and the larger symbols represent their averages. The squares correspond to the two-point correlation length. For *ϕ* = 0.76, the asymptotic slope was used in calculating *ξ*_d_

Analogous to the behavior of static correlations in liquids with externally introduced pins^20,21,23^, the excess contribution to the configurational overlap over the bulk, |*q*_∞_(*r*)−*q*_rand_|, decays exponentially with *r* for all *ϕ*'s even for self-induced pins (Fig. 2a). This allowed us to estimate a static correlation length *ξ*_stat_ from the relation |*q*_∞_(*r*)−*q*_rand_| = *B*exp(−*r*/*ξ*_stat_). As seen in simulations and experiments, we observe that the prefactor *B* depends on *ϕ*^20,21^. We explicitly account for this dependence by defining a second static length scale *ξ*_stat−int_ = *Bξ*_stat_^20^. We next attempted to extract the dynamic length scale *ξ*_d_. In Fig. 2b, we show ${\rm ln}(\tau _{\mathrm{s}}(r)/\tau _{\mathrm{s}}^{{\mathrm{bulk}}})$ versus *r* for different *ϕ*'s. For all *ϕ*'s except *ϕ* = 0.76 (inverted red triangles in Fig. 2b), ${\rm ln}(\tau _{\mathrm{s}}(r)/\tau _{\mathrm{s}}^{{\mathrm{bulk}}})$ shows an exponential decay. For *ϕ* = 0.76, which is close to the mode-coupling area fraction (*ϕ*_MCT_ = 0.77, Supplementary Fig. 4), however, we clearly see two slopes. A similar departure from an exponential decay has been observed in the presence of a pinned amorphous wall^20,21^ and was attributed to the presence of multiple relaxation processes near the MCT transition^19^. We extract *ξ*_d_ from the relation ${\rm ln}(\tau _{\mathrm{s}}(r)/\tau _{\mathrm{s}}^{{\mathrm{bulk}}}) = B_{\mathrm{s}}{\mathrm{exp}}( - r/\xi _{\mathrm{d}})$^20^.

We perform the equivalent of disorder averaging by repeating the above analysis for atleast 6–8 pins(≈12 pins are used in calculating *ξ*_d_ for *ϕ* = 0.75−0.79) within our field of view. We ensure that the average inter-pin separation is >10–12*σ*_s_. The small hollow triangles and circles in Fig. 2c correspond to *ξ*_stat_ and *ξ*_d_ evaluated for each of these pins and the larger symbols correspond to their averages for each *ϕ* (Supplementary Fig. 5). We also show the two-point correlation length *ξ*_*g*(*r*)_ (hollow green squares) obtained by fitting an envelope to the decay of the pair-correlation function *g*(*r*) for comparison. While *ξ*_stat_ appears to grow faster than *ξ*_*g*(*r*)_, the more striking feature is the presence of a maximum in *ξ*_d_. The observed trends are not very sensitive to the value of *q*_c_(*τ*_*α*_) used to define a self-induced pin or issues with sample equilibration beyond *ϕ*_MCT_ (Supplementary Note 2 and Supplementary Figs. 6–10). This is the first observation of a non-monotonic evolution of a dynamic length scale in a bulk supercooled liquid. The presence of this maximum near *ϕ*_MCT_ is consistent with the change in shape of dynamical heterogeneities observed earlier^21^. Also, both *ξ*_stat_ and *ξ*_d_ for self-induced pins grow weaker than the amorphous wall geometry^21^. While this could simply be due to the lack of disorder averaging in the latter, simulations find that the length scales in the random pinning geometry, where the pin configuration is similar to the self-induced pinning case, evolve much more slowly than for other pin geometries^23^ and are also consistent with theoretical predictions^25^. Further, while the standard deviation in *ξ*_stat_ remains nearly same for all *ϕ*'s studied, for *ξ*_d_ it is maximal near *ϕ*_MCT_ (Supplementary Fig. 11) and this may be another signature of being in the vicinity of the dynamical crossover predicted by RFOT. More importantly, these observations unambiguously show that the non-monotonic evolution of *ξ*_d_, hitherto observed only in liquids in the presence of a pinned amorphous wall^20,21^, is clearly a feature of the bulk liquid and is not an outcome of the pinning procedure as alluded in recent studies^29,31^.

### Identifying amorphous–amorphous interfaces

**Fig. 3:**
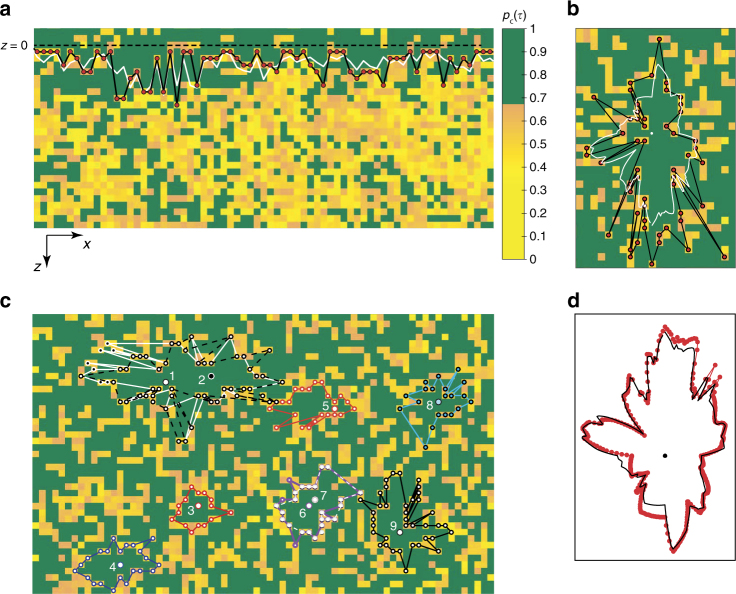
Identifying amorphous–amorphous interfaces. **a** The background image represents the *p*_c_(*τ*_*α*_) for a portion of the field of view containing the amorphous wall located at *z* ≤ 0 (dashed line) for *ϕ* = 0.75. The color bar represents *p*_c_(*τ*_*α*_) values. The black line through the red symbols corresponds to the instantaneous interface profile *h*(*z*,*t*) and the white line is the time-averaged profile 〈*h*(*z*,*t*)〉_*t*_. **b** Interface profile around self-induced pin represented by the white circle. The background color scheme and the lines have the same meaning as in **a**. **c** Instantaneous interface profiles around distinct self-induced pins (represented by the numbered circles). Pins 1 and 2 and pins 6 and 7 lie within the same mosaic and yield nearly the same interface. **d** Instantaneous interface profile post fuzzy-grid averaging. The black line represents the 〈*h*(*z*,*t*)〉_*t*_ around the pin (black circle). In **b**–**d**, *ϕ* = 0.79

The striking similarities in the evolution of static and dynamic length scales between artificially introduced and self-induced pinning motivated us to explore if the methods for identifying amorphous–amorphous interfaces in the former can be extended to the latter^13–15^. Since experimental studies of such interfaces are lacking even for pinned liquids, we first analyze data on colloidal liquids in the presence of an optically pinned amorphous wall^21^ (Supplementary Note 3 and Supplementary Fig. 12). We coarse-grain the field of view into 1*σ*_s_ boxes and subsequently calculate the persistence function *p*_c_(*t* = *τ*_*α*_) = 〈*p*_*i*_(*t*)〉, where *p*_*i*_(*t*) = 1 if the occupancy of box *i* remains unchanged at time *t* = 0 and *t* = *t* and *p*_*i*_(*t*) = 0 otherwise^27^. The 〈〉 denotes averaging over 1*τ*_*α*_. This minor modification to the definition of *q*_c_(*t*) ensures that, post coarse-graining, an empty box in an immobile region of the liquid is treated on par with an occupied one. Figure 3a shows a snapshot of *p*_c_(*τ*_*α*_) for *ϕ* = 0.75. The image corresponds to the portion of the field of view that contains the wall located at *z* ≤ 0 (dashed line). In line with expectations, *p*_c_(*τ*_*α*_) for regions close to the wall (*z* > 0) is larger as compared to regions farther away from it (represented by green and yellow boxes, respectively). Next, starting from each box at *z* = 0, we scan along *z* and locate the box where the overlap dropped to *p*_c_(*τ*_*α*_) ≤ 0.67. The line joining these boxes delineates the regions of high and low configurational overlap^14,15^ and we define this to be the instantaneous interface profile, *h*(*z*,*t*), for a given *τ*_*α*_ (black line in Fig. 3a). We follow this procedure for each *τ*_*α*_ and quantify the dynamics of *h*(*z*,*t*) (Supplementary Note 4). We find that the time-averaged interface profile, 〈*h*(*z*,*t*)〉_*t*_, as expected, is parallel to the wall at *z* = 0 (white line in Fig. 3a). For each *ϕ*, interface fluctuations are probed over their corresponding *τ*_*α*_ and a direct comparison of their dynamics is thus possible. Supplementary Movies 1 and 2 show interface fluctuations for the pinned wall geometry for *ϕ* = 0.75 and *ϕ* = 0.79, respectively.

We made modifications to the above procedure for identifying interfaces around self-induced pins. Starting from the pin (represented as solid white circle in Fig. 3b), we scan along *x* and *z* directions for boxes where the overlap dropped to *p*_c_(*τ*_*α*_) ≤ 0.67 (Supplementary Note 4 and Supplementary Fig. 13). A line through these boxes represents the instantaneous interface profile. Figure 3c shows the interfaces obtained from this procedure for many well-separated pins within our field of view for *ϕ* = 0.79. At high *ϕ*'s especially, the regions with a high configurational overlap typically contain more than one self-induced pin (Supplementary Fig. 2). We have checked that the interface profile obtained starting from any of these pins defines nearly the same high overlap region (white and pink interface profiles around pins labeled 1 and 2 and 6 and 7, respectively, in Fig. 3c). We then carry out the fuzzy-grid averaging procedure to smoothen out interface fluctuations for both the pinned wall and self-induced pins. The resulting interface profile is interpolated (black line through red symbols in Fig. 3d) before further analysis. Supplementary Movies 3 and 4 show interface fluctuations around self-induced pins for *ϕ* = 0.75 and *ϕ* = 0.79, respectively.

### Growing surface tension of amorphous–amorphous interfaces

**Fig. 4:**
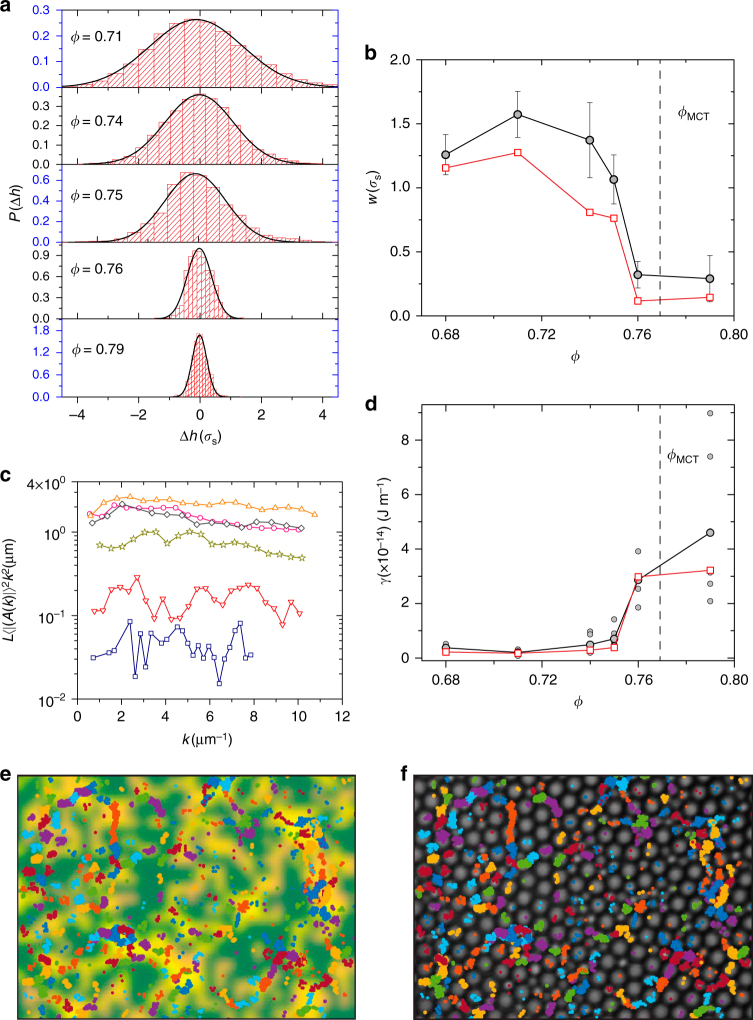
Surface tension of amorphous–amorphous interfaces. **a** Normalized histogram of height fluctuations for various *ϕ*s. The black lines represent Gaussian fits to the data. **b** Interface width *w* versus *ϕ* for amorphous wall (hollow squares) and for self-induced pins (hollow circles). Error bars represent the standard deviation of *w* obtained from distinct self-induced pins. **c** *L*〈|*A*(*k*)|^2^〉*k*^2^ versus *k* for a representative self-induced pin. *ϕ* = 0.68 (pink circles), *ϕ* = 0.71 (orange triangles), *ϕ* = 0.74 (gray diamonds), *ϕ* = 0.75 (dark yellow stars), *ϕ* = 0.76 (red inverted triangles), and *ϕ* = 0.79 (blue squares). **d** The small hollow circles represent *ϒ* for distinct self-induced pins and the large circles represent their average for each *ϕ*. *ϒ* versus *ϕ* for amorphous wall (hollow squares). **e** The background image represents *p*_c_(*t* = 7t*) for *ϕ* = 0.79. **f** Snapshot of liquid configuration corresponding to **e**. In **e** and **f**, the trajectories of the top 1% most-mobile particles are shown by the colored symbols

The surface tension of an interface is inversely related to its roughness, which is best captured by the interface width $w = \sqrt {(\Delta h)^2} = \sqrt {(h(z,t) - \langle h(z,t)\rangle _t)^2}$^37–41^. Since the amplitude of interface fluctuations is extensive in the system size and diverges in the thermodynamic limit, we only consider interfaces of the same length (Supplementary Note 5). Figure 4a and Supplmentary Fig. 14a show the evolution in the distribution of height fluctuations, *P*(Δ*h*) with *ϕ*, for the self-induced pin and the pinned wall, respectively. *P*(Δ*h*) gets narrower with *ϕ* which already signals a growth in the *ϒ* of the high *p*_c_ regions. While *P*(Δ*h*) is well captured by Gaussian fits at low *ϕ*'s, we observe deviations for *ϕ*>0.75 and hence extract *w* by fitting a Gaussian only to the central region where *P*(Δ*h*) dropped by about a decade. In Fig. 4b, we show *w* versus *ϕ* for the self-induced pin (solid symbols) and the pinned wall (hollow symbols). Strikingly, for both these cases, we observe that *w* appears to taper off beyond *ϕ*_MCT_.

Next, we attempt to measure *ϒ* directly using the CFM. CFM was originally developed to quantify the dynamics of flat interfaces separating phases with a well-defined order parameter^38–41^, and it is not immediately apparent if this approach can be extended to configurational overlap fields and that too for interfaces that are curved like those seen around self-induced pins. At finite temperature, interfaces undergo broadening due to thermal fluctuations and the final equilibrium profile is a trade off between the surface energy term which prefers a flat interface and the thermal energy *k*_B_*T*. In CFM, *h*(*z*,*t*) is decomposed into normal modes and the amplitude of each mode decays as 〈|*A*(*k*)|^2^〉 = *k*_B_*T*/*Lϒk*^2^ in accordance with the equipartition theorem. Here, *k* is the wavevector and *L* is the length of the interface. Figure 4c and Supplementary Fig. 14b show *L*〈|*A*(*k*)|^2^〉*k*^2^ versus *k* as a function of *ϕ* for a representative self-induced pin and the pinned wall, respectively. We find that in both cases, *L*〈|*A*(*k*)|^2^〉*k*^2^ is constant for almost a decade in *k* which not only validates the applicability of CFM for the present system but also allowed us to quantify *ϒ* for the first time in experiments. Our findings are not very sensitive to the precise definition of the configurational overlap used, the coarse-graining box size, and the choice of time scale in defining the interface (Supplementary Notes 6 and 7 and Supplementary Figs. 15–21). Figure 4d shows *ϒ* versus *ϕ* for many well-separated self-induced pins (small gray circles) and the pinned wall (hollow squares). The filled black circles correspond to the average *ϒ* for each *ϕ*. Remarkably, we observe that with supercooling, *ϒ* grows rather rapidly in the vicinity of *ϕ*_MCT_.

In the original version of RFOT^5^, the concept of a mosaic state is not meaningful beyond the spinodal singularity where metastability is lost (*T* > *T*_c_ regime), and hence *ϒ* is also expected to drop to zero above *T*_c_ (*ϕ*<*ϕ*_MCT_ in colloid experiments). In our experiments, we not only find *ϒ* to be finite but it also grows as *ϕ*_MCT_ is approached from below (Supplementary Fig. 22). A generalized RFOT, proposed recently^13,16^, suggests that the value of *ϒ* between distinct pairs of amorphous mosaics is not identical, as assumed earlier^5^, but is instead a broad distribution with only the average *ϒ* vanishing for *T* ≥ *T*_c_^13^. A broad distribution in *ϒ* implies that the mosaic state can survive in the *T* > *T*_c_ regime and the sharp transition at *T*_c_ is thus smeared out. Theoretical arguments also anticipate that the distribution of activation energy barriers develop long tails near *T*_c_, due to presence of secondary string-like relaxation processes, resulting in a smoothening of the transition^43^. Thus, while a non-zero value of *ϒ* even at the lowest *ϕ*'s we study may simply be an outcome of our analysis procedure, the systematic growth in *ϒ* lends support to the generalized RFOT. Strikingly, the experimentally observed growth in *ϒ* is in accord with the expected^19^ and observed compaction of cooperatively relaxing regions (CRRs) on supercooling across *ϕ*_MCT_^20,21^.

### Anisotropic caging at amorphous–amorphous interfaces

We finally focus on the dynamics of most-mobile particles at amorphous–amorphous interfaces. In Fig. 4e, we show the top 1% of particles that were labeled most-mobile over a t* interval^44^ that fell within the time window (7t*) over which the underlying *p*_c_(*t*) was calculated for *ϕ* = 0.79. Figure 4f shows a snapshot of the liquid at the beginning of the 7t* window. The different colors in Fig. 4e and f represent different particles. While it is not surprising that these particles are predominatly found in the low *p*_c_ regions, we observe that for most particles the trajectories are elongated along the interface length. This anisotropic weakening of the cage should result in string-like CRRs oriented along the interface and is strikingly similar to the dynamics of particles at crystal grain boundaries^45^.

## Discussion

The novel scheme put forth here for identifying self-induced pins has helped unveil the existence of interfaces separating adjacent relaxing regions in bulk supercooled liquids. Although our experiments are restricted to the boundary of the RFOT regime ($\phi \sim \phi _{{\mathrm{MCT}}}$) in order to avoid too large relaxation times, the observed rapid growth in the surface tension with supercooling is in line with theoretical expectations of a crossover between null amorphous surface tension and a finite one. Further, the non-monotonic evolution in *ξ*_d_ with *ϕ* finds a natural explanation only within the RFOT paradigm. Crucially, the fact that the static and dynamical length scales and interface dynamics in the bulk liquid mirror those seen in the pinned liquid strengthens numerous findings on the latter which favor a thermodynamic origin of the glass transition. Given that the involved procedure of artificially pinning particles is not essential to verify RFOT opens the door to extending our method to supercooled liquids made of particles with complex shapes and internal degrees of freedom. In the ongoing quest for identifying the relevant length scale(s) that best capture the growth in *τ*_*α*_ in the *T* < *T*_c_ (*ϕ* > *ϕ*_MCT_) regime^29^, determining whether *ξ*_d_ is eventually slaved to *ξ*_stat_ is crucial. While this is presently beyond the scope of particle-resolved experiments, numerical studies that exploit the swap Montecarlo technique may be the way forward^46,47^. CRRs are a generic feature of other competing theoretical frameworks of the glass transition as well and whether our observations can be reconciled within these approaches remains an open challenge. Finally, the very existence of such amorphous–amorphous interfaces opens up the possibility of tuning their properties, and hence that of the glass itself, in a manner analogous to grain boundary engineering in polycrystals^48^.

## Methods

### Experimental details

Our experimental system comprises of a binary mixture of colloidal polystyrene particles with sizes *σ*_s_ = 1.05 μm and *σ*_L_ = 1.4 μm, respectively. The number density ratio of big and small particles *N*_L_/*N*_S_ = 1.23 is sufficient to prevent crystallization and is held nearly constant for all area fractions, *ϕ*, studied. Here, *ϕ* plays the role of an inverse temperature. Single-particle dynamics are studied using optical video microscopy. The colloidal suspensions are loaded in a wedge-shaped cell that is left standing for a suitable time duration to yield the desired particle area fraction *ϕ*. The systems are equilibrated for a typical time duration of 8–10 h before the experiments (several times *τ*_*α*_ for all *ϕ*s <0.79). Samples are imaged using a Leica DMI 6000B optical microscope with a 100× objective (Plan apochromat, NA 1.4, oil immersion) and images are captured at frame rates ranging from 3.3 fps to 5 fps for 1–1.5 h depending on the values of *ϕ*. The typical field of view captured in our experiment is of the size 72*σ*_s_ × 44*σ*_s_. The analysis reported here is carried out on experiments that  were performed immediately after the holographic optical tweezers, that are used to pin an amorphous wall of particles, was turned off^21^ (Supplementary Note 3). Thus, a direct comparison of the present findings with the earlier study is justified. The particle trajectories are obtained from standard MATLAB algorithms^49^. Subsequent analysis is performed using codes developed in-house. The typical drift observed in the experimental data in the presence of a pinned amorphous wall is of the order of 0.7*σ*_s_ in the *x*-direction and 0.15*σ*_s_ in the *z*-direction over the entire duration of the experiment. Since sample drift corrections are not possible in the presence of the wall, we choose the longest time window where the sample drift was <0.1*σ*_s_ for further analysis.

### Data availability

The data that support the findings of this study are available from the corresponding author upon request.

## Electronic supplementary material

Supplementary Information

Description of Additional Supplementary Files

Supplementary Movie 1

Supplementary Movie 2

Supplementary Movie 3

Supplementary Movie 4
