# Supplementary material for: Measurements of growing surface tension of amorphous–amorphous interfaces on approaching the colloidal glass transition
Source: Nat Commun. 2018 Jan 26;9:397. doi: 10.1038/s41467-018-02836-6 (PMC5786034; doi:10.1038/s41467-018-02836-6)
Supplement: Supplementary file 1 — Supplementary Information [file 41467_2018_2836_MOESM1_ESM.pdf]

SUPPLEMENTARY FIGURES

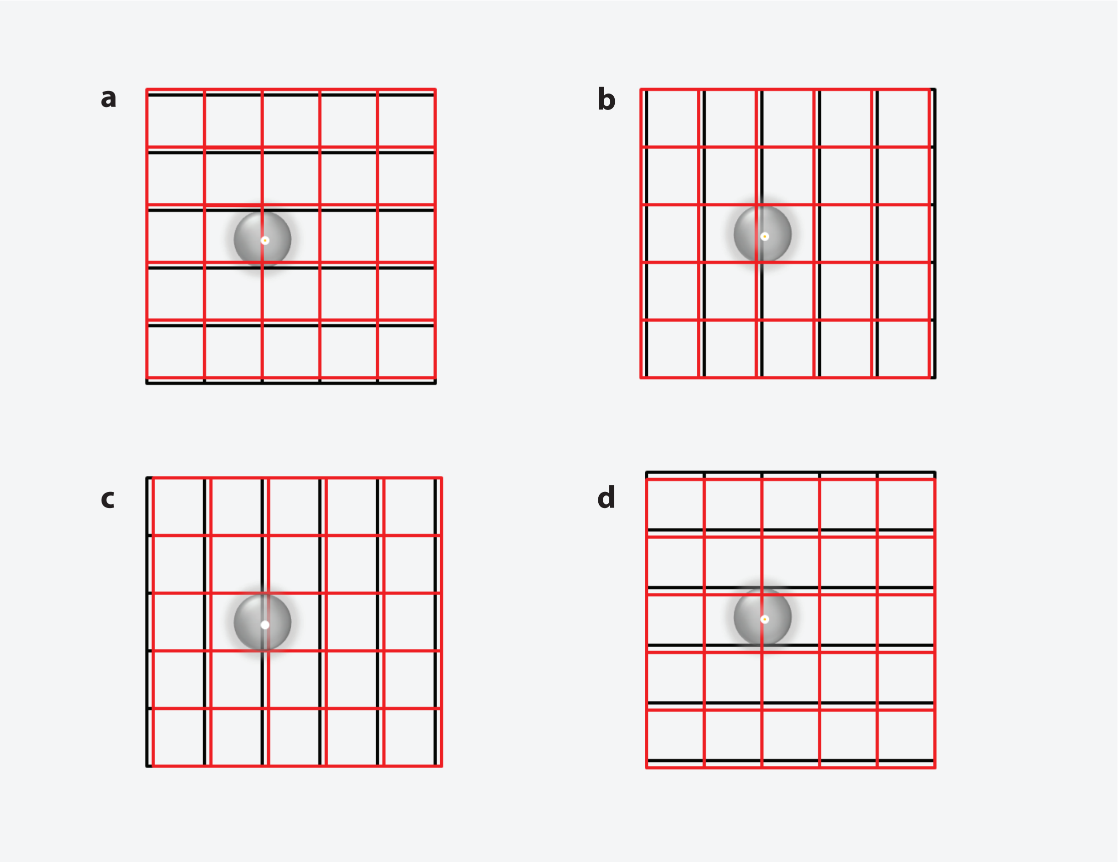

Supplementary Figure 1 | Fuzzy-Grid Method. Black and red grids represent original and displaced coarse grained configurations respectively. The white dot represents the particle position. The grid configurations represented in red are displaced by an amount $0.1\sigma_{\mathrm{s}}$ along +Y-axis in **a**, -X-axis in **b**, +X-axis in **c** and -Y-axis in **d**.

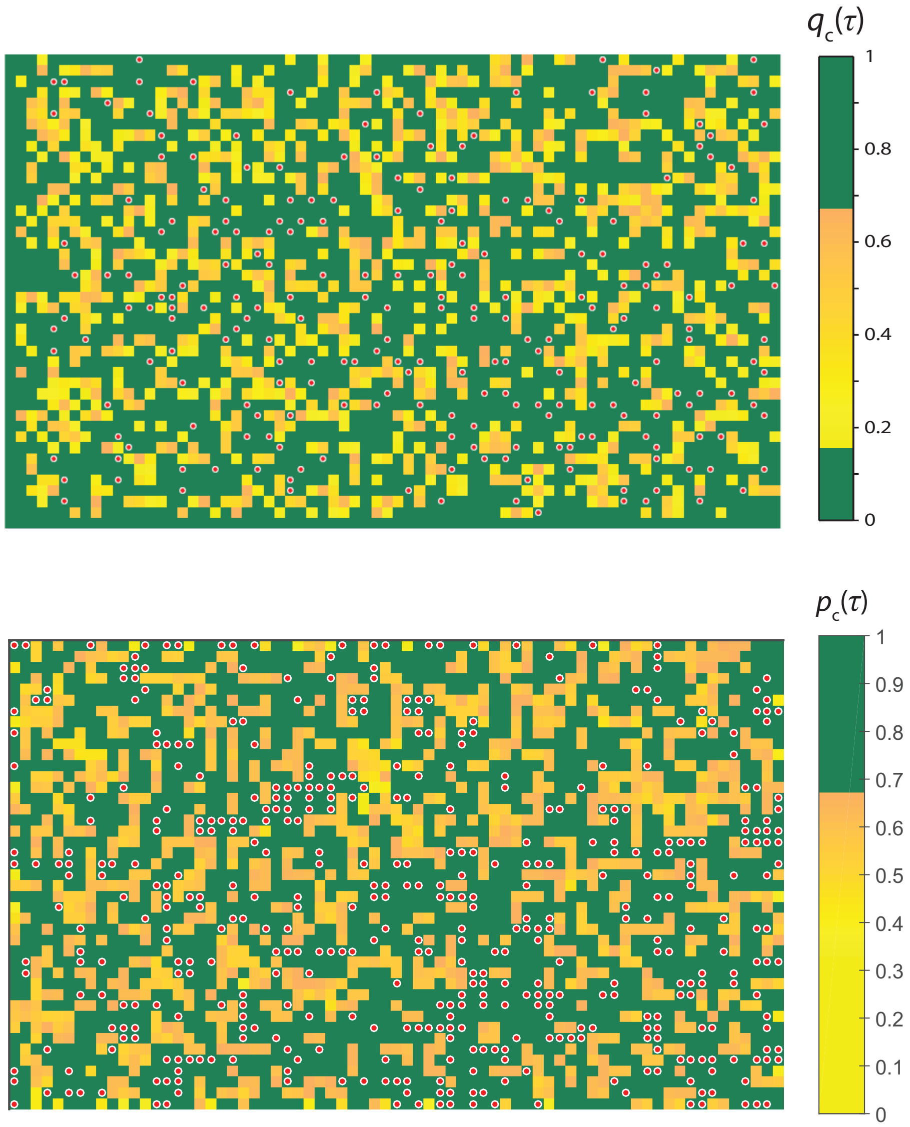

Supplementary Figure 2 | Self induced Pins for ϕ=0.79. (a) The background image represents $q_c(t)$ for the full field of view for ϕ=0.79 over the whole experimental duration. The coarse-graining size is $1\sigma_s$. The filled white circles represent self-induced pins which stayed put for the entire experimental duration. The color bar represents the value of $q_c(t)$. Green and yellow represent high and low configurational overlaps, respectively. (b) The background image represents $p_c(t)$ for the full field of view for ϕ=0.79 over the whole experimental duration. The coarse-graining size is $1\sigma_s$. The filled white circles represent self-induced pins which stayed put for the entire experimental duration. The color bar represents the value of $p_c(t)$. Green and yellow represent high and low persistence, respectively.

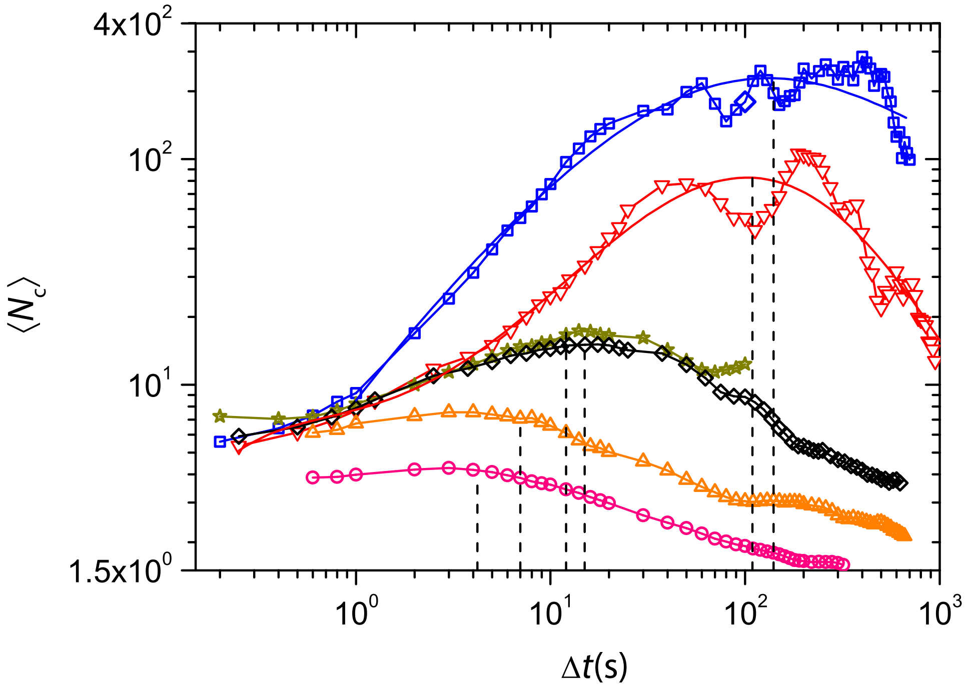

Supplementary Figure 3 | Evolution of average cluster size of the top 10% most-mobile particle clusters with ϕ. ϕ=0.68 (pink circles), ϕ=0.71 (orange triangles), ϕ= 0.74 (gray diamonds), ϕ=0.75 (dark yellow stars), ϕ=0.76 (red inverted triangles) and ϕ=0.79 (blue squares). The time at which the cluster size is a maximal was taken to be the cage-breaking time t^* (Reproduced with permission from [2]).

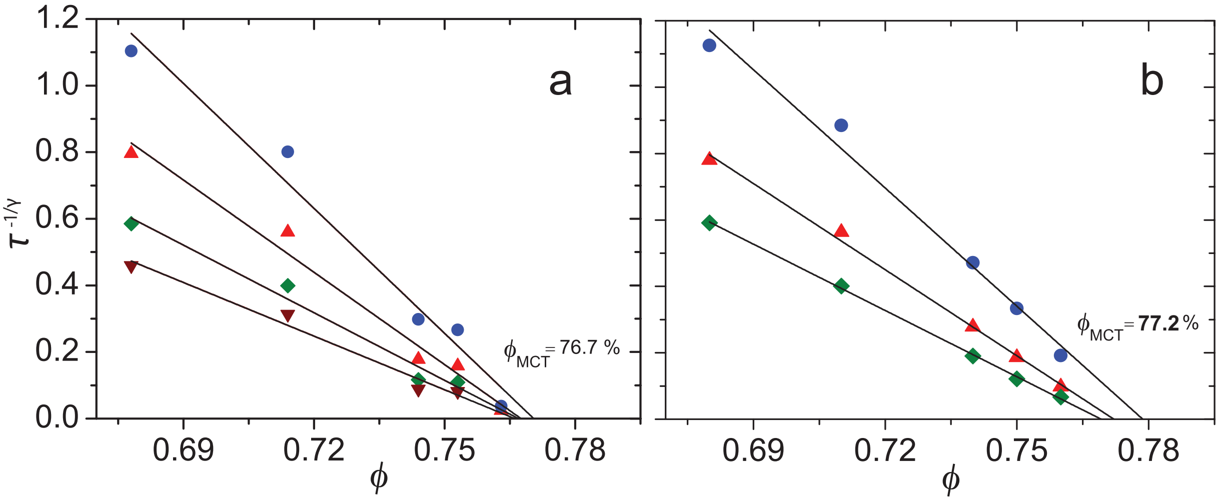

Supplementary Figure 4 | Estimation of mode-coupling crossover area fraction, $\phi_{\mathbf{MCT}}$. $\tau^{\frac{-1}{\gamma}}$ versus ϕ for wave vectors $q = 2\pi/0.75\sigma$ (navy circles),$q = 2\pi/\sigma$ (red triangles) and $q = 2\pi/1.25\sigma$ (olive green diamonds) where σ is the mean diameter of the big and small particles. Here,$\gamma=\frac{1}{2a}+\frac{1}{2b}$=1.89 has been computed by using a=0.377, obtained from ref [1,2,3] and b=0.885, extracted from $F_s(q,t)$. (a) $\tau^{\frac{-1}{\gamma}}$ versus ϕ for data in the absence of pinning. (b) $\tau^{\frac{-1}{\gamma}}$ versus ϕ for the region far away from the pinned wall. (Reproduced with permission from [2]).

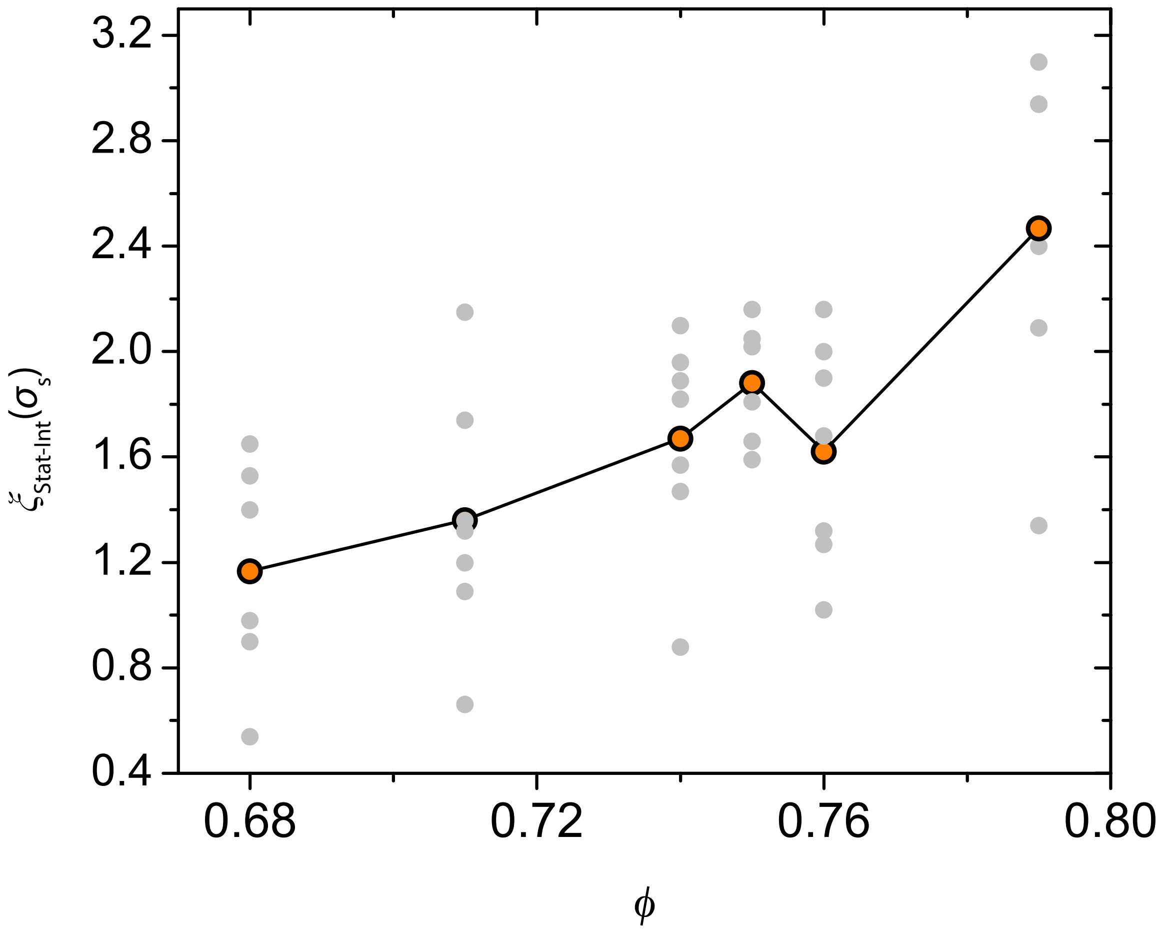

Supplementary Figure 5 | $\xi_{\textbf{stat-int}}$ versus ϕ The grey circles correspond to $\xi_{\text{stat-int}}$ for distinct pins and the black circles represent the average $\xi_{\text{stat-int}}$ for each ϕ. $\xi_{\text{stat-int}}$ was observed to grow systematically with supercooling.

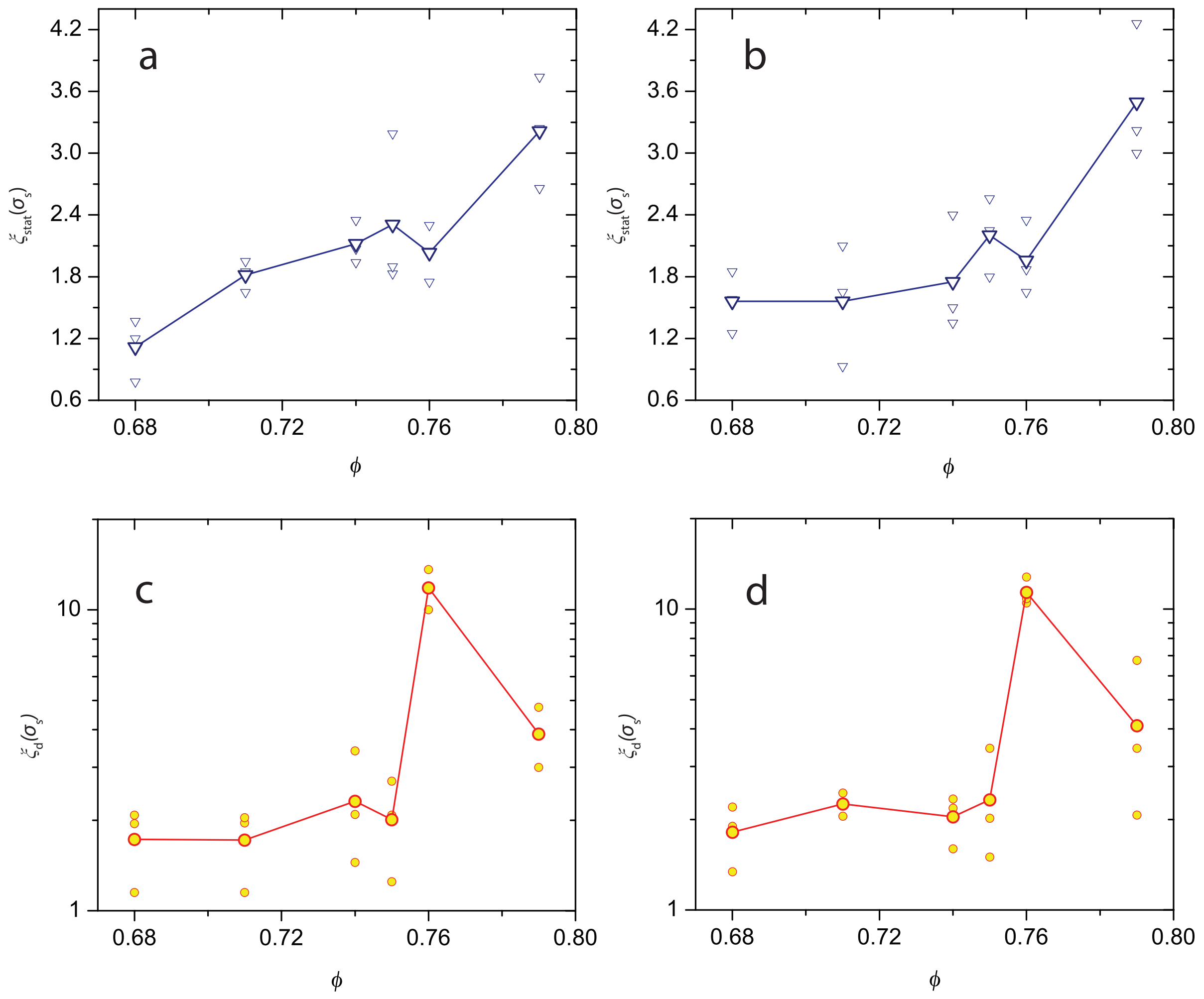

Supplementary Figure 6 | Static and dynamic length scales for different cut-off values used in defining self-induced pins (a) and (b) show evolution of ξ_{stat} with ϕ for $q_{\mathrm{c}}(\tau_\alpha) = 0.85$ and 0.95, respectively. (c) and (d) show evolution of ξ_{d} with ϕ for $q_{\mathrm{c}}(\tau_\alpha) = 0.85$ and 0.95, respectively.

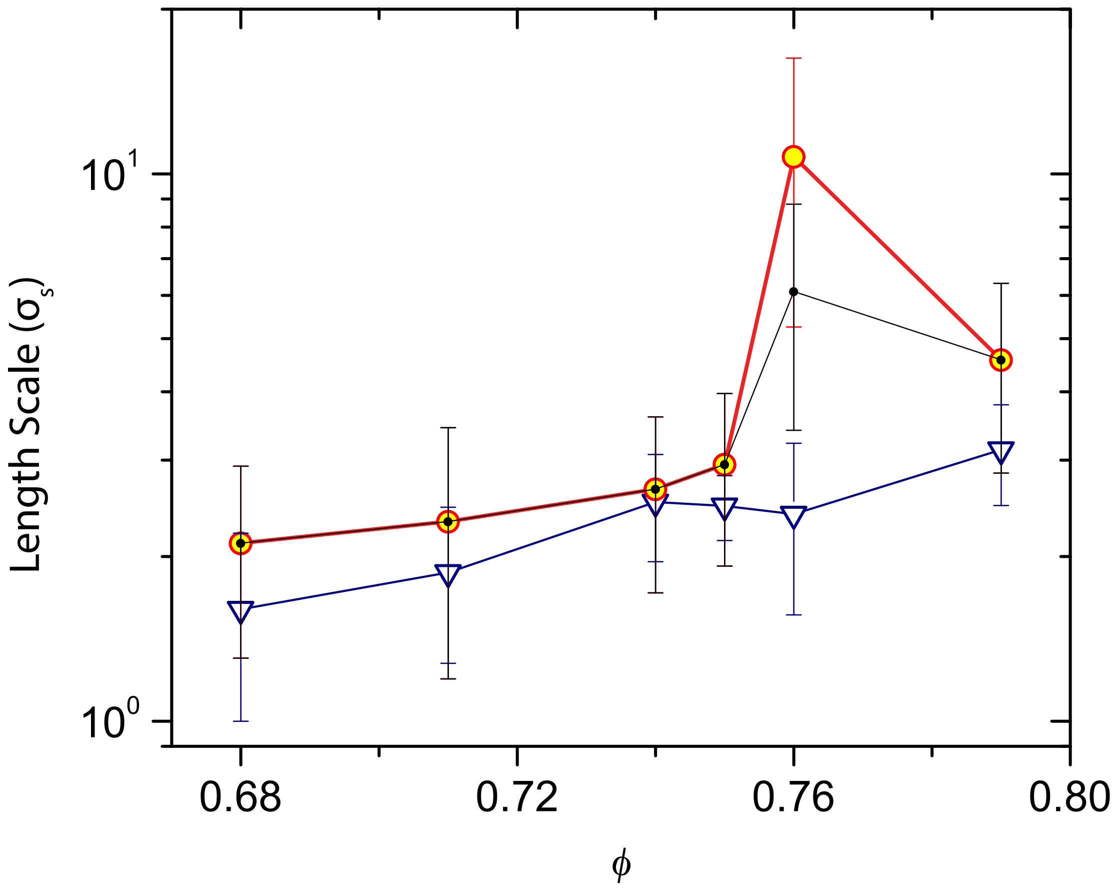

Supplementary Figure 7 | Estimation of errors in $\xi_{\mathbf{stat}}$ and $\xi_{\mathbf{d}}$ The yellow circles and blue triangles represent ξ_{d} and ξ_{stat}, respectively. The errors represent the standard deviation of the mean obtained from repeating the analysis for multiple self-induced pins. The black circles correspond to ξ_{d} obtained from force fitting a single slope to $\ln(\tau_{\mathrm{s}}(r)/\tau_{\mathrm{s}}^{\mathrm{bulk}})$ versus ϕ for $\phi = 0.76$. For all other ϕ's we observe only a single slope.

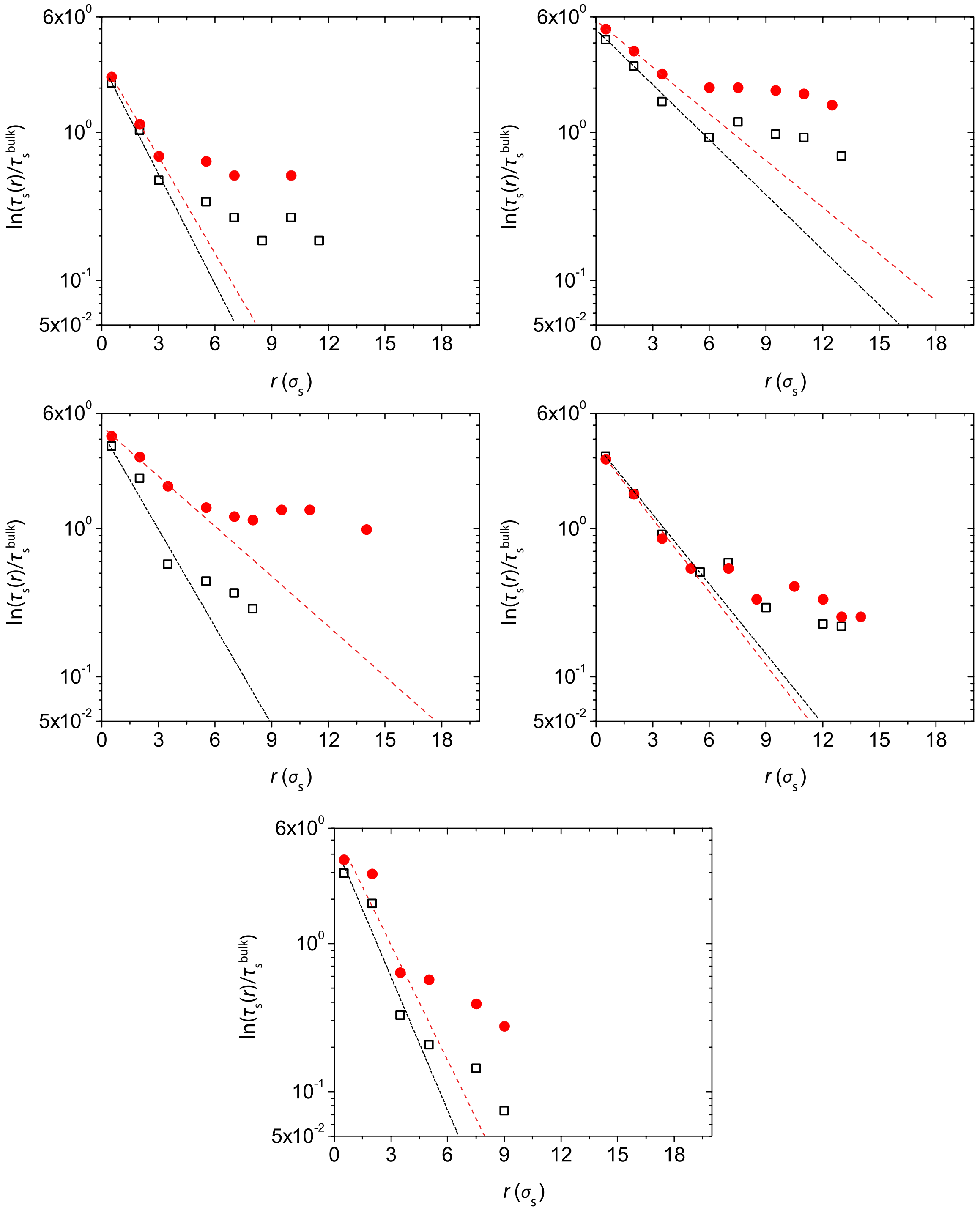

Supplementary Figure 8 | Effect of under equilibration on dynamics for $\phi = 0.76$. Panels a-e show $\ln(\tau_s(r)/\tau_s^{\text{bulk}})$ for $\phi = 0.76$ for distinct pins. The red symbols correspond to the full experimental duration ($> \tau_\alpha$) and the black symbols for the partial data ($\approx 0.5\tau_\alpha$). The red and black lines are only a guide to the eye to highlight the deviation from a single slope at large r.

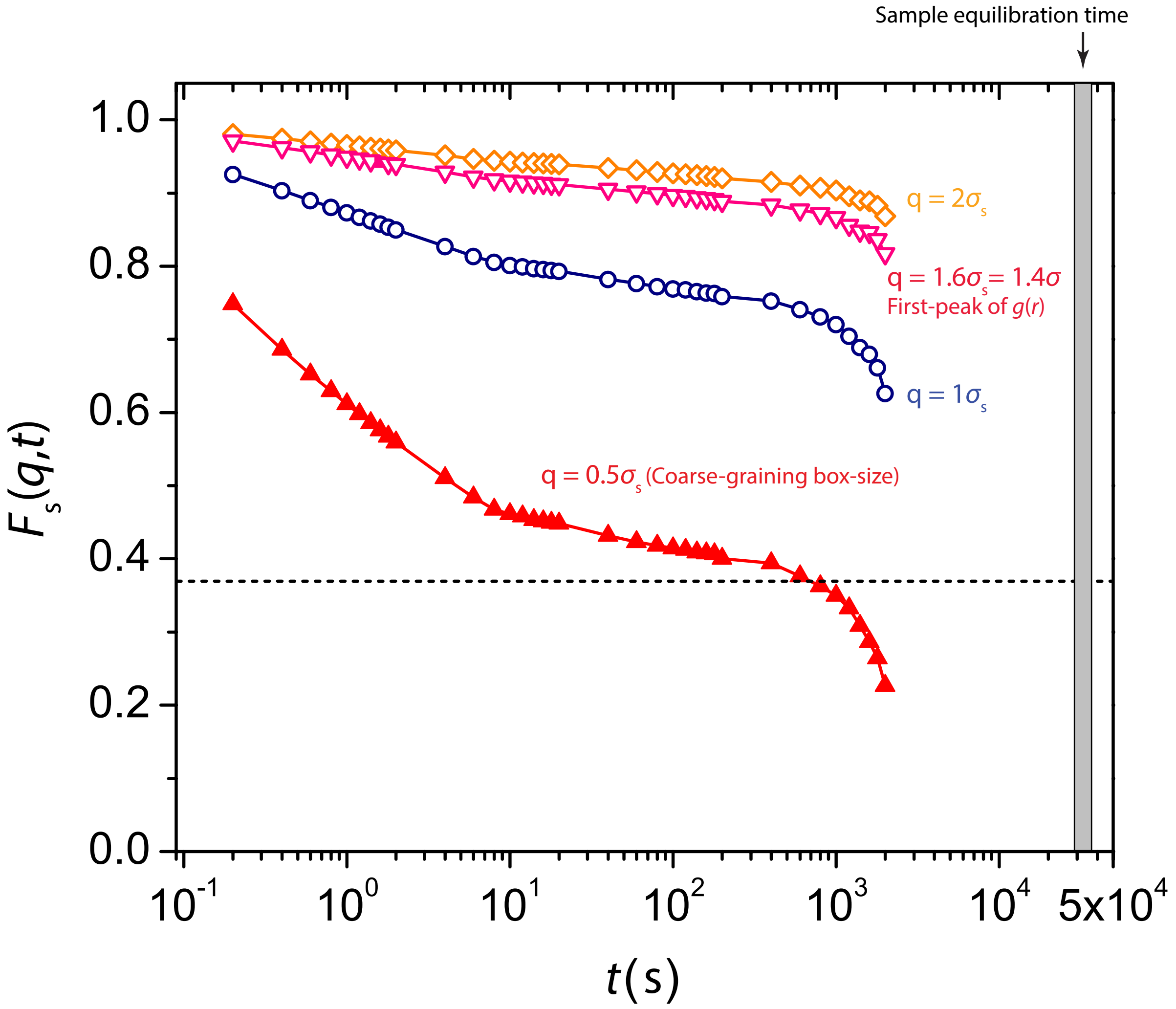

Supplementary Figure 9 | **$F_s(q,t)$ for $\phi = 0.79$ for various q's.** The dashed horizontal line corresponds to $1/e$ and the vertical gray shaded region denotes the sample equilibration time before data collection.

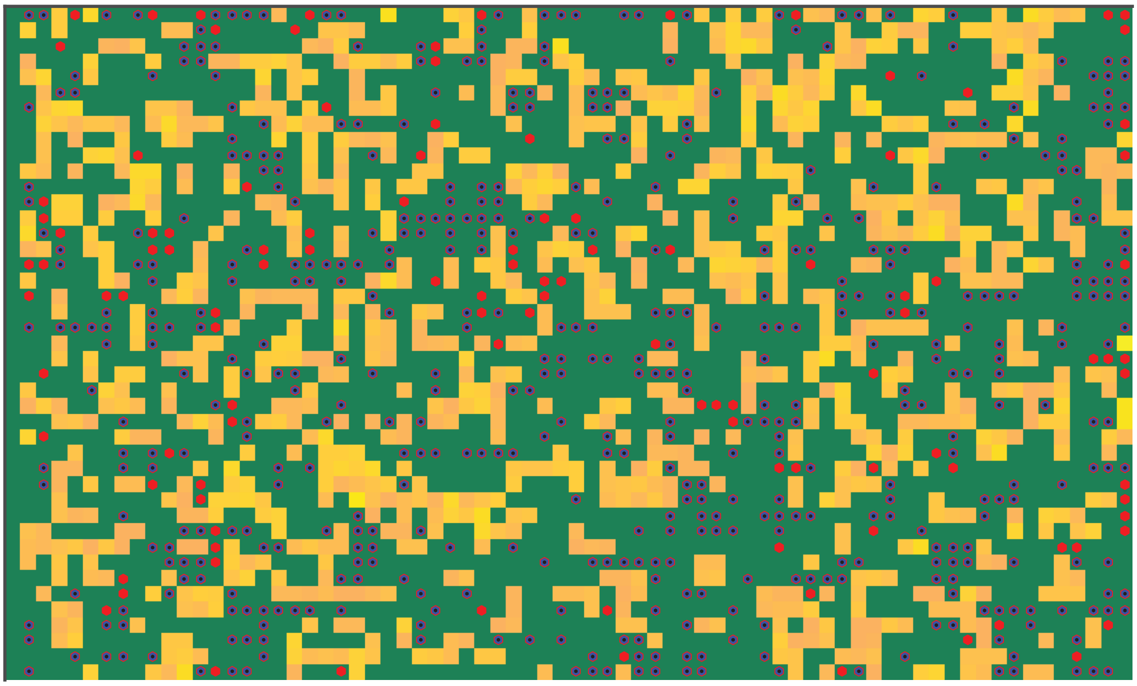

Supplementary Figure 10 | Evolution of Self-induced pins with time. The red symbols were pins that survived from $t = 0$ to $t = 3t^*$ and the blue symbols represent the subset of pins that survived until $t = 7t^*$.

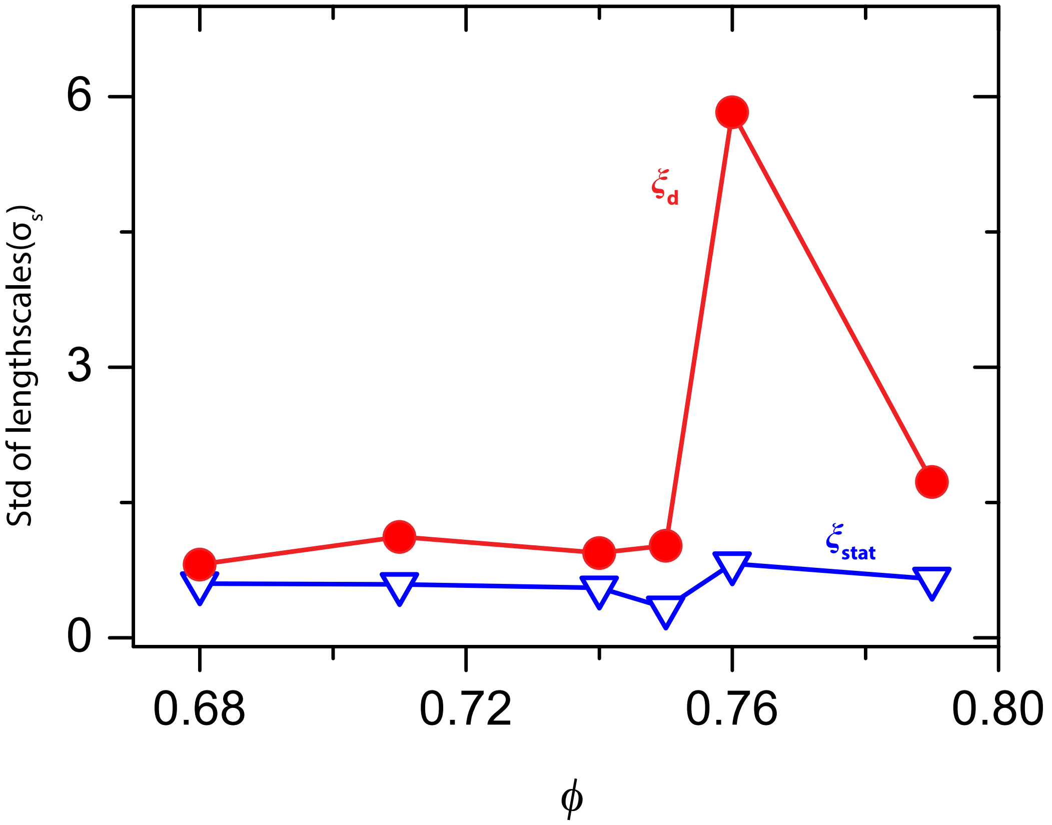

Supplementary Figure 11 | Standard deviation of length scales. Standard deviation in the static correlation length, $\xi_{\rm stat}$(blue down triangles) and the dynamic correlation length, $\xi_{\rm d}$(red circles) obtained from various self-induced pins considered in Fig. **2c** of the main text.

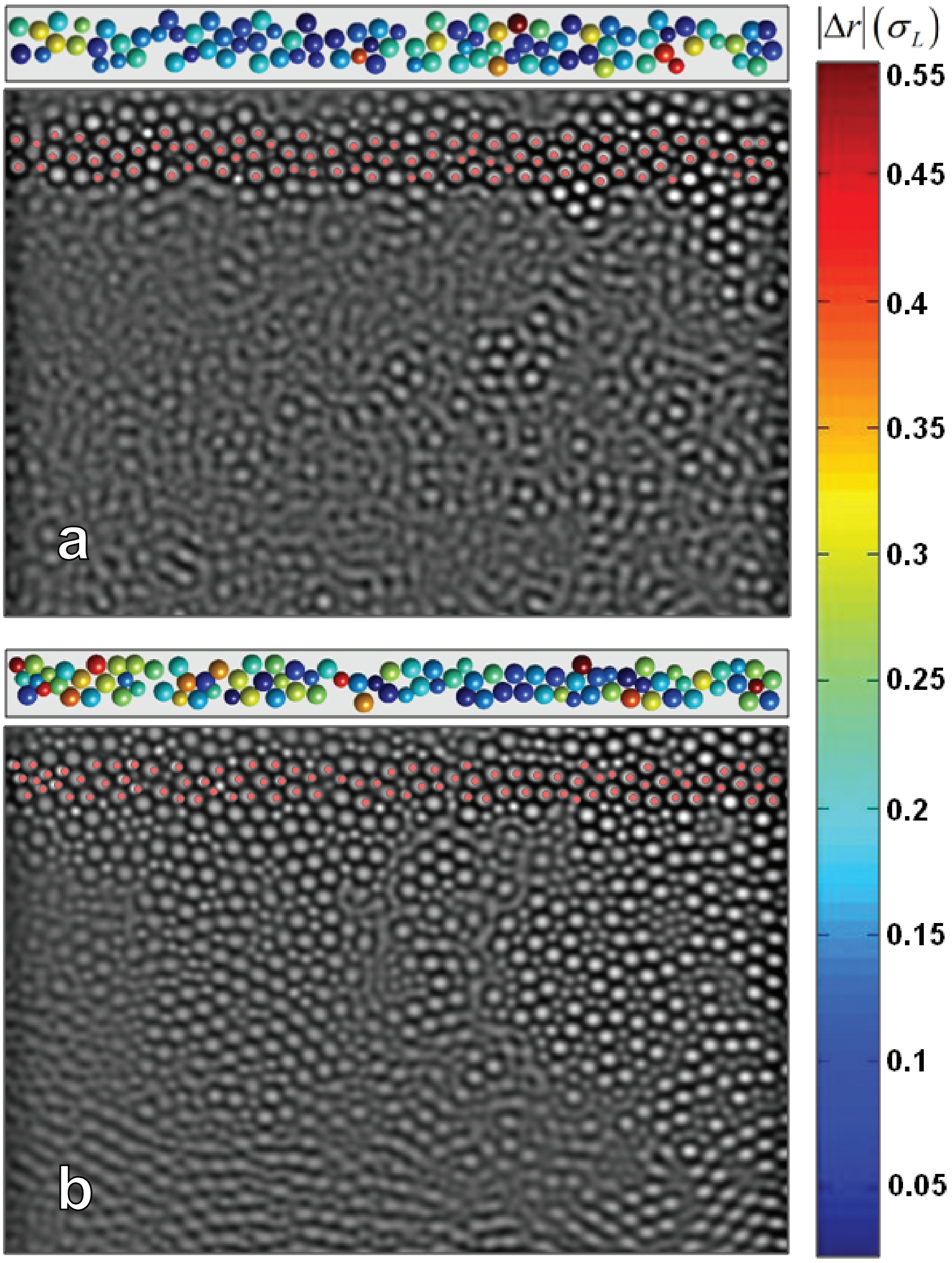

Supplementary Figure 12 | Visualization of the amorphous wall. The underlying grey scale images have been generated by time-averaging snapshots over $30\tau_\alpha$ for ϕ=0.68 (**a**) and ϕ=0.76 (**b**) for a pinned wall. τ_α=12s and 274s for **a** and **b**, respectively. The red circles correspond to the coordinates of the trapped particles that form the amorphous wall. The spheres at the top of the images in **a** and **b** constitute the pattern whose fast Fourier transform was fed into the spatial light modulator. Spheres are colour coded according to the distance between the input coordinates for creating traps and time-averaged particle positions in units of σ_{L}.(Reproduced with permission from [2]).

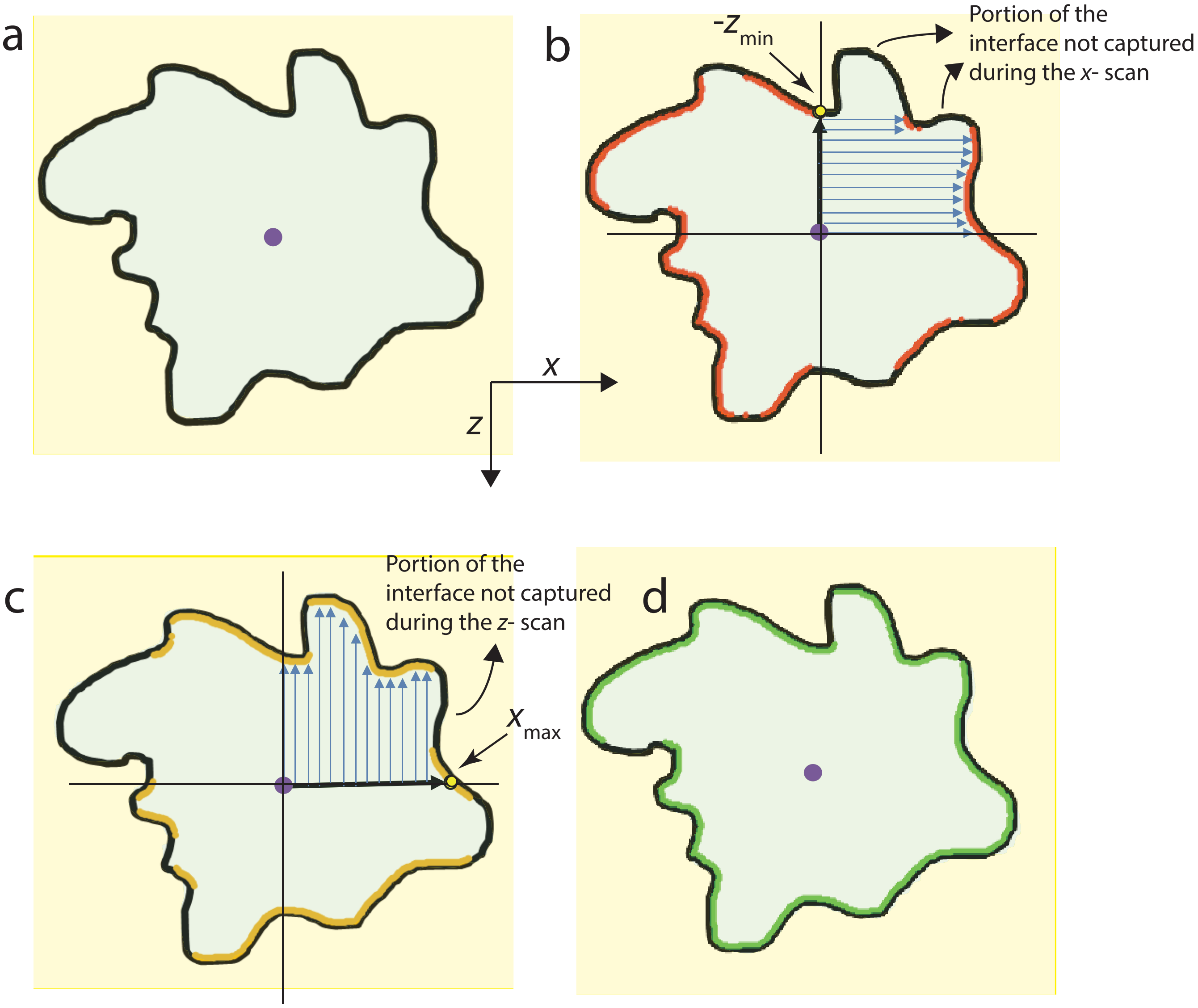

Supplementary Figure 13 | Procedure for identifying interfaces around self-induced pin. a Artificially generated interface (black line) delineating regions of high (green) and low (yellow) configurational overlap. **b** The scan along -z-axis terminates at $-z_{\rm min}$ on encountering a low overlap box and is shown as a thick black arrow. The blue arrows represent scans along the x direction and terminate at the interface (shown in red). **c** Shows scans along direction orthogonal to those shown in **b**. **d** The procedure is repeated for all quadrants and the final interface profile (shown in green) is generated.

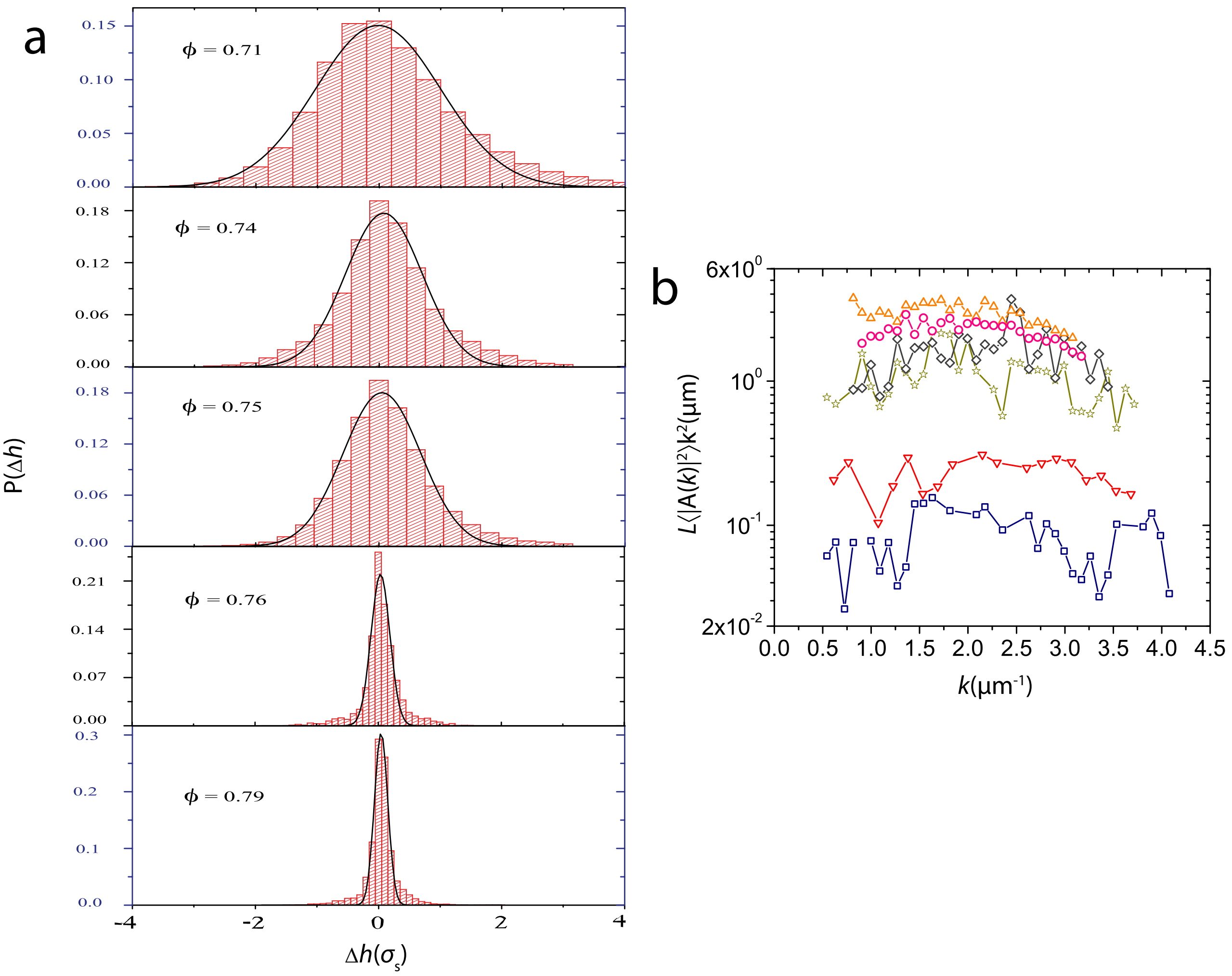

Supplementary Figure 14 | Height fluctuations and $L\langle|A(k)|^2\rangle k^2$ versus k for amorphous wall configuration.(a) Normalized histogram of height fluctuations for various ϕs. The black lines represent Gaussian fits to the data. (b) $L\langle|A(k)|^2\rangle k^2$ for ϕ=0.68 (pink circles), ϕ=0.71 (orange up triangles), ϕ= 0.74 (gray diamonds), ϕ=0.75 (dark yellow stars), ϕ=0.76 (red down triangles) and ϕ=0.79 (blue squares).

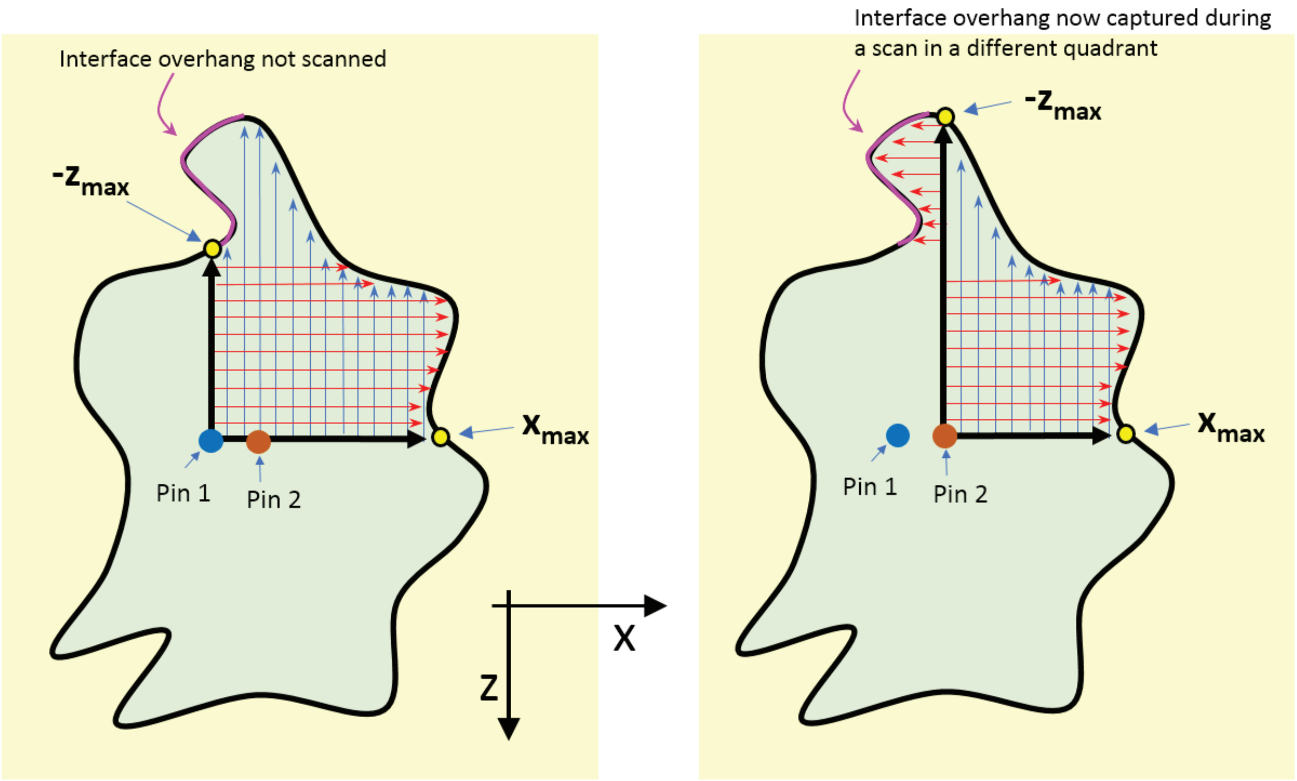

Supplementary Figure 15 | Influence of interface overhangs on calculation of surface tension a Artificially generated interface (black line) delineating regions of high (green) and low (yellow) persistence. Starting from Pin 1, the purple portion of the interface remains unscanned during both the x and z scans. **b** Starting from Pin 2, the overhang is accessible during a scan in a different quadrant.

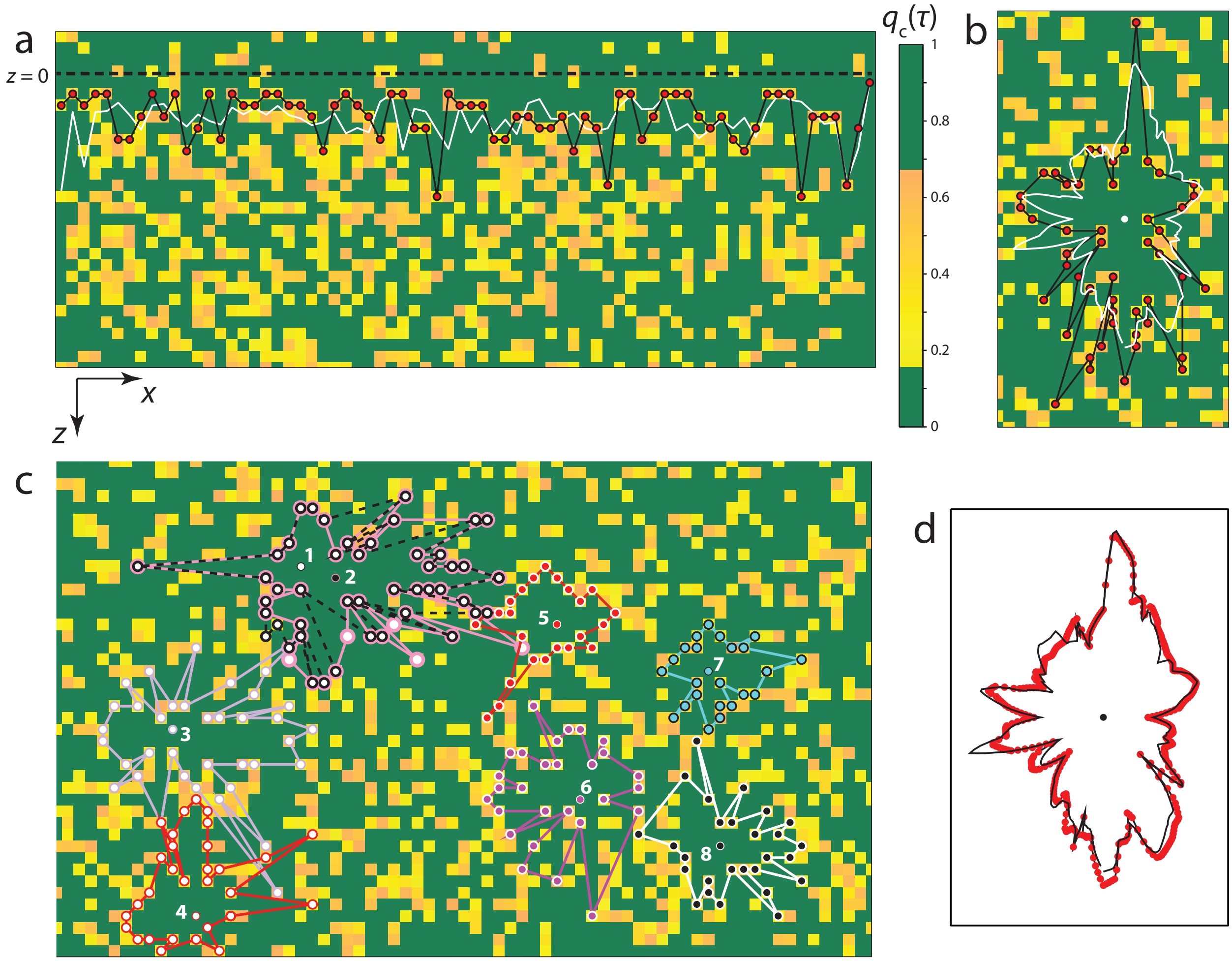

Supplementary Figure 16 | Identifying Amorphous-amorphous interfaces using q_c. (a) The back ground image represents the $q_c(\tau_\alpha)$ for a portion of the field of view containing the amorphous wall located at $z \leq 0$ (dashed line) for $\phi = 0.75$. The color bar represents $q_c(\tau_\alpha)$ values. The black line through the red symbols corresponds to the instantaneous interface profile $h(z, t)$ and the white line is the time-averaged profile $\langle h(z, t) \rangle_t$. (b) Interface profile around self-induced pin represented by the white circle. The background color scheme and the lines have the same meaning as in (a). (c) Instantaneous interface profiles around distinct self-induced pins (represented by the numbered circles). Pins 1 and 2 lie within the same mosaic and yield nearly the same interface. (d) Instantaneous interface profile post fuzzy-grid averaging. In (b)-(d) $\phi = 0.79$.

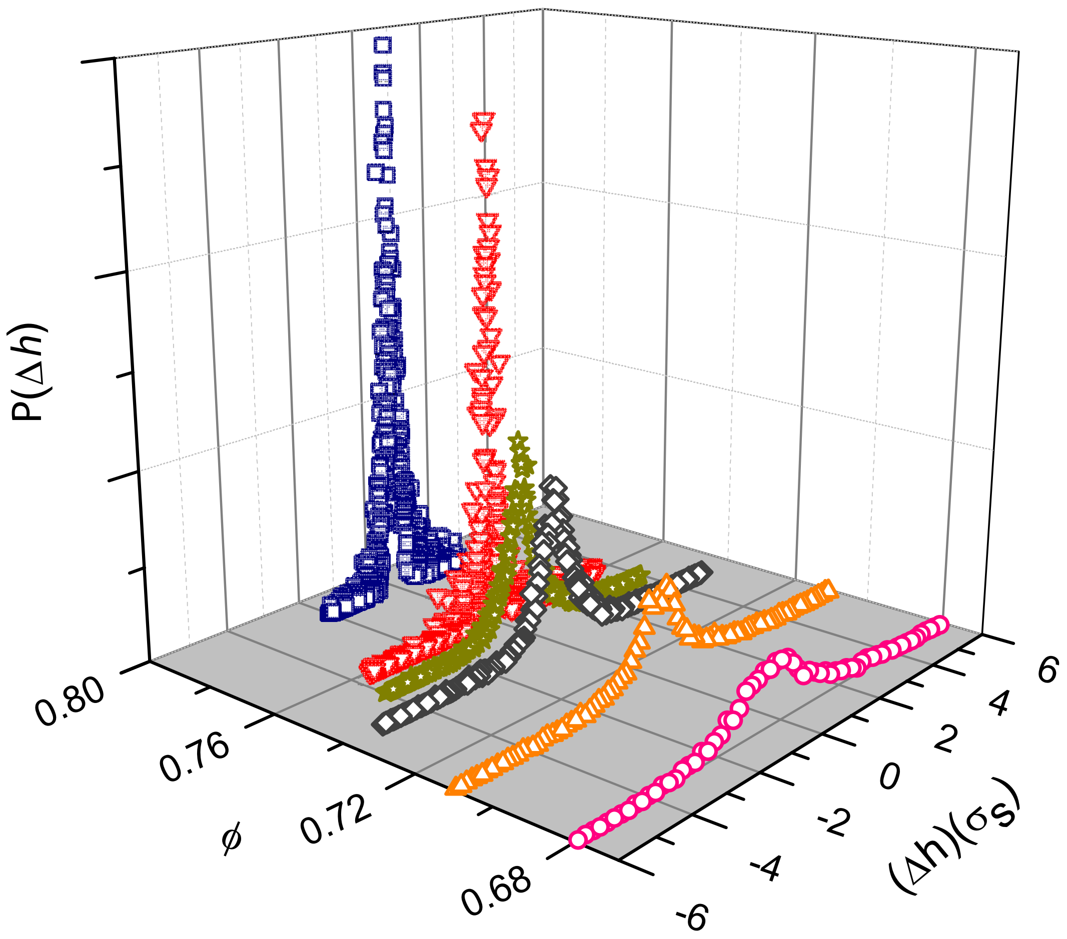

Supplementary Figure 17 | Probability distribution of height fluctuations *P*(Δ*h*) for the pinned amorphous wall. *P*(Δ*h*) for ϕ=0.68 (pink circles), ϕ=0.71 (orange up triangles), ϕ= 0.74 (gray diamonds), ϕ=0.75(dark yellow stars), ϕ=0.76 (red down triangles) and ϕ=0.79 (blue squares). Fluctuations for all ϕs except $\phi = 0.79$ are calculated over 1.4 τ_α for comparison. For, $\phi = 0.79$, due to issues with sample equilibration $t = 10t^*$.

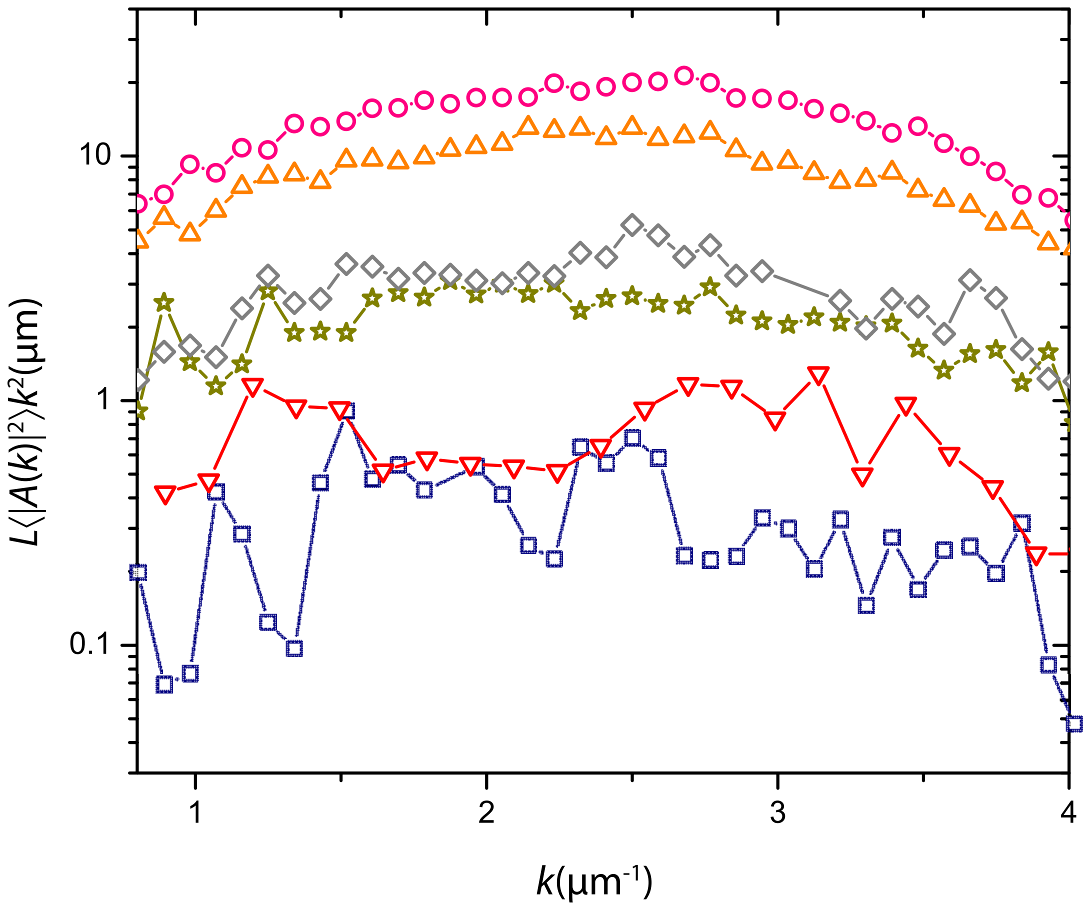

Supplementary Figure 18 | $L\langle|A(k)|^2\rangle k^2$ versus k for amorphous wall configuration. $L\langle|A(k)^2|\rangle k^2$ for ϕ=0.68 (pink circles), ϕ=0.71 (orange up triangles), ϕ= 0.74 (gray diamonds), ϕ=0.75 (dark yellow stars), ϕ=0.76 (red down triangles) and ϕ=0.79 (blue squares).

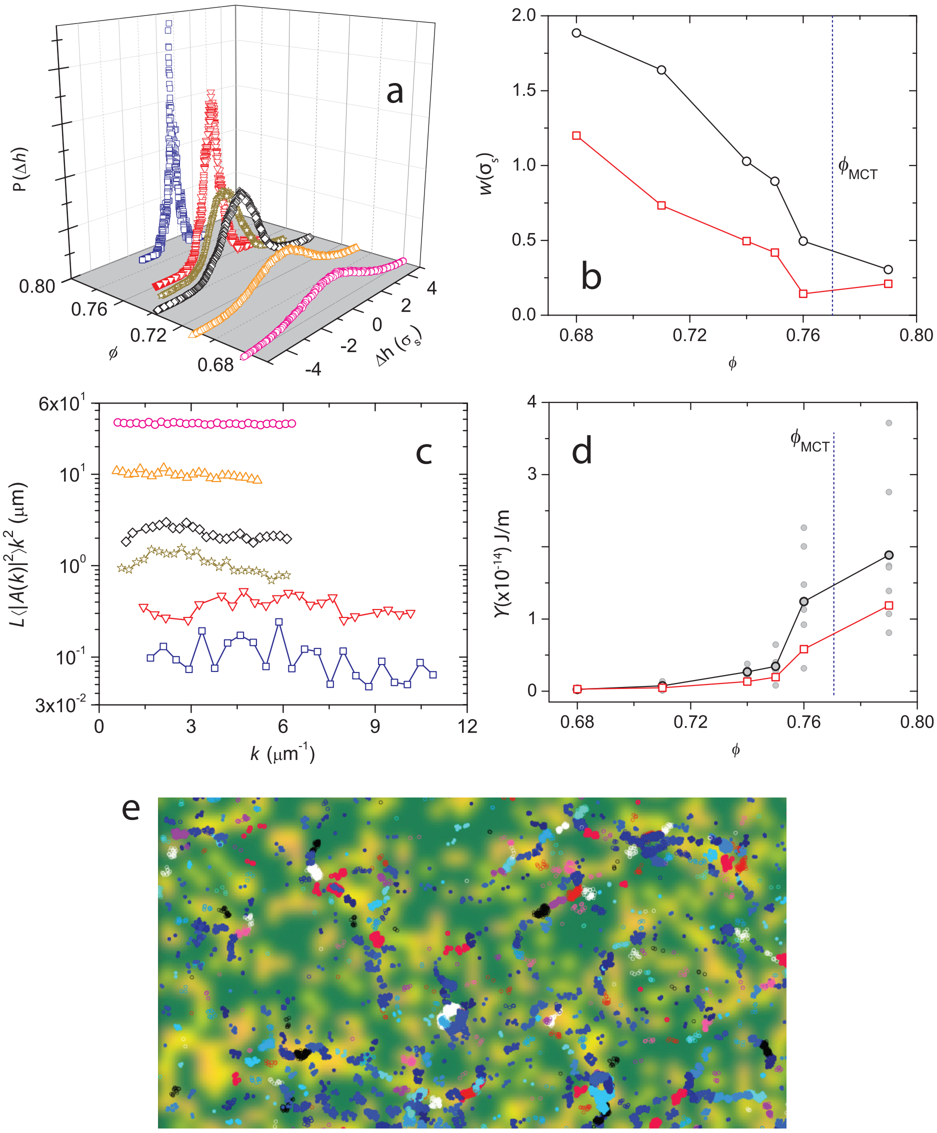

Supplementary Figure 19 | Surface tension of amorphous-amorphous interfaces using $q_{\mathbf{c}}$. (a) Distribution of height fluctuations as a function of ϕ. ϕ=0.68 (pink circles), ϕ=0.71 (orange up triangles), ϕ= 0.74 (gray diamonds), ϕ=0.75(dark yellow stars), ϕ=0.76 (red down triangles) and ϕ=0.79 (blue squares). (b) Interface width w versus ϕ for amorphous wall (hollow squares) and for a representative self-induced pin (hollow circles). (c) $L\langle|A(k)|^2\rangle k^2$ versus k for a representative self-induced pin. The symbol shape correspond to same ϕ's as in (a). (d) the small hollow circles represent Υ for distinct self-induced pins and the large circles represents their average for each ϕ. Υ versus ϕ for amorphous wall (hollow squares). (e) The background image represents $q_c(t = 7t^*)$ for $\phi = 0.79$. The trajectories of the top 1% most-mobile particles are shown by the colored symbols.

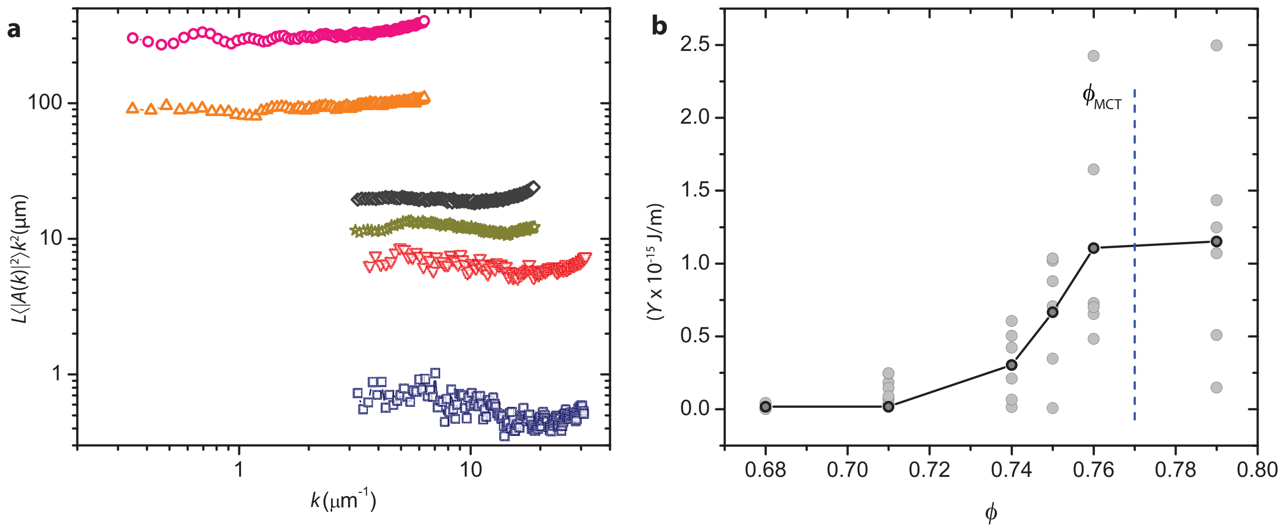

Supplementary Figure 20 | Surface tension measurements of interfaces around self-induced pins for grid size of 0.5σ_s. a, $L\langle|A(k)|^2\rangle k^2$ versus k for ϕ=0.68 (pink circles), ϕ=0.71 (orange up triangles),ϕ= 0.74 (gray diamonds), ϕ=0.75 (dark yellow stars), ϕ=0.76 (red down triangles) and ϕ=0.79 (blue squares) for a representative self-induced pin. **b**, Surface tension (Υ) as a function of ϕ. (Gray filled circles) represents surface tension values for well-separated pins at each ϕ, (black open circles) represents their average value at every ϕ.

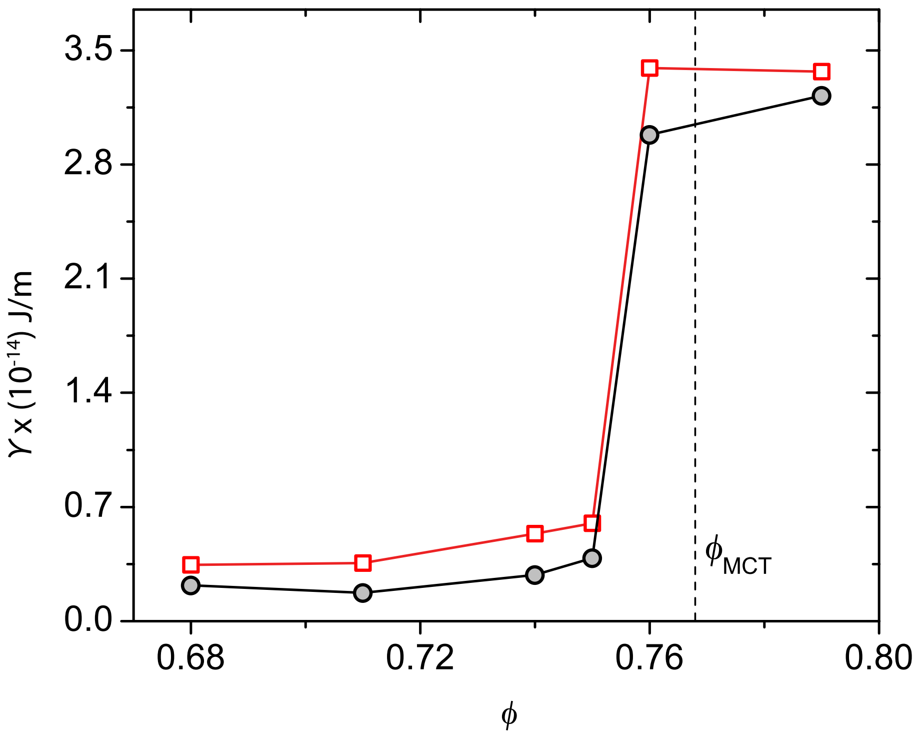

Supplementary Figure 21 | Υ versus ϕ for a pinned amorphous wall for different time scales used in defining the interface. The circles correspond to interfaces that are defined over τ_α for $\phi \leq 0.76$ and $7t^*$ for $\phi = 0.79$ from the p_c analysis. The squares correspond to interfaces that are defined over a larger time scale ($3\tau_\alpha$ for $\phi < 0.76$, $2\tau_\alpha$ for $\phi = 0.76$ and $14t^*$ for $\phi = 0.79$) also from the p_c analysis.

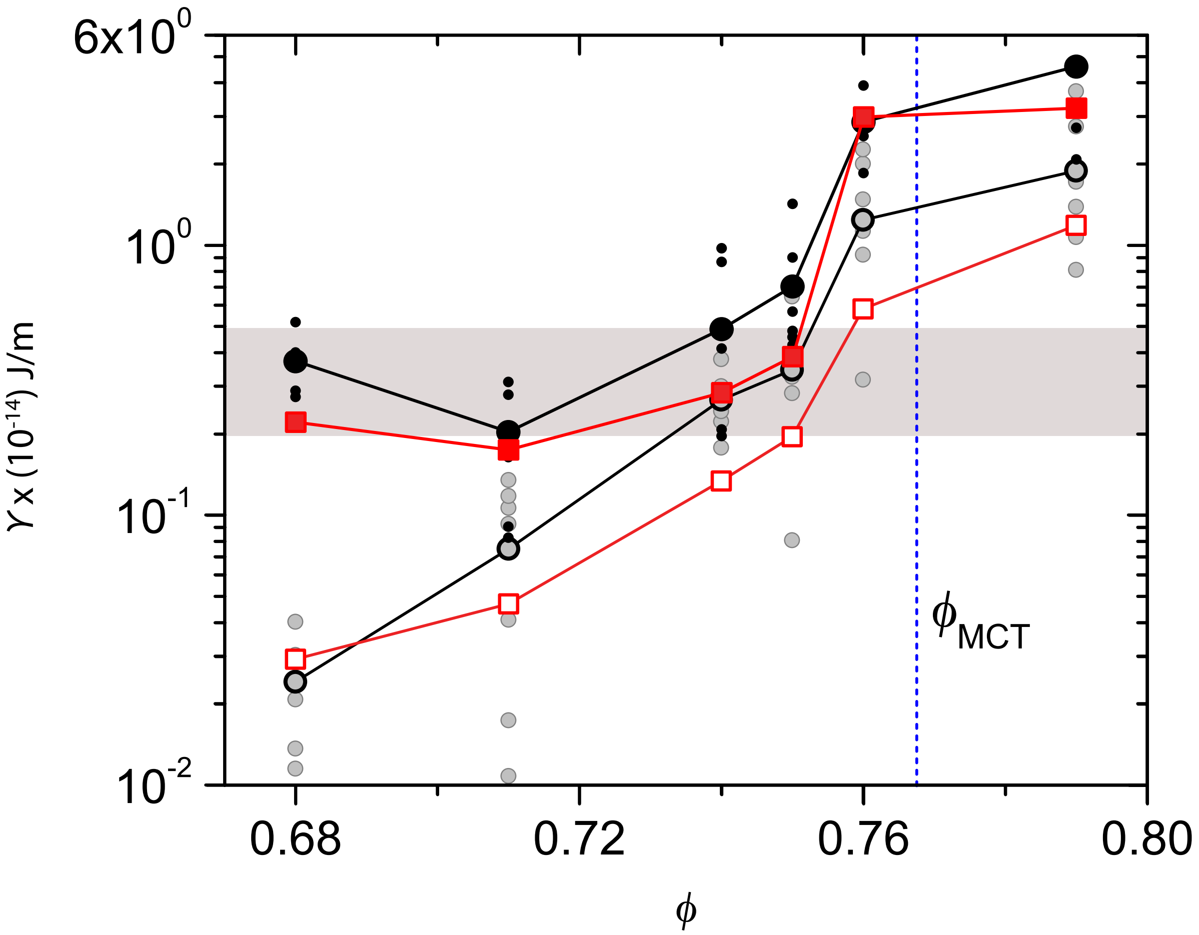

Supplementary Figure 22 | Log-Linear representation of Υ versus ϕ. The solid and hollow symbols represent Υ obtained from p_c and q_c analysis, respectively. Results for the amorphous wall and self-induced pins are represented by squares and circles, respectively. The small circles represent Υ for distinct self-induced pins and the large circles represents their average for each ϕ. The shaded region is a guide to the eye to show that Υ from the p_c analysis appears to saturate at low ϕ.

SUPPLEMENTARY NOTES

Supplementary Note 1 | Fuzzy-Grid method.

To calculate the configurational overlap $q_{\mathrm{c}}(t)$ or the persistence function $p_{\mathrm{c}}(t)$ (see section **Identifying amorphous-amorphous interfaces** of the main manuscript), we coarse grain the field of view into boxes of size $1\sigma_{\mathrm{s}}$. A particle with its centre close to a box edge can hop between neighboring boxes leading to spurious fluctuations in $q_{\mathrm{c}}(t)$ and $p_{\mathrm{c}}(t)$(Supplementary Fig. 1). This effect is particularly pronounced for boxes whose overlap value is near the cut-offs chosen to identify interfaces. Thus, considering a rigid box configuration leads to selecting those boxes that may be unrelated to the true position of the interfaces. In order to account for fluctuations due to such events, we displace the original grid configuration for the whole field of view by $0.1\sigma_{\mathrm{s}}$ ($\approx$ cage size) in different directions (along $\pm$X axis, $\pm$Y axis and the diagonal of the four quadrants). Further, a coarse grained box is considered to be a pin only when its $q_{\mathrm{c}} > 0.9$ or $p_{\mathrm{c}} > 0.9$, as the case may be, in all the grid configurations realized. (Supplementary Fig. 2) represents pins which are present for the whole duration of the experiment for ϕ=0.79. At low ϕs, the self-induced pins considered stay put for a minimum of 3-5τ_α. For $\phi = 0.76$, the pins stay put for the total experimental duration ($2.5\tau_\alpha$) and for $\phi = 0.79$, the pins stay put for the total experimental duration ($18t^*$).

Supplementary Note 2 | Influence of sample equilibration on the dynamic length scale $\xi_{\mathbf{d}}$

In our experiments, for all ϕs we investigate, the samples were equilibrated for 8-10hrs before data collection. Subsequently, data is typically collected for 1-1.5 hrs at frame rates ranging from 3-5 fps. Owing to experimental limitations, arising primarily from temperature and mechanical drifts, we are unable to do longer experiments. Such drifts are a bane of particle-resolved colloid experiments and the means to completely avoid them continue to remain a challenge. While for $\phi < \phi_{\mathrm{MCT}}$, the experimental duration is much larger than τ_α, for $\phi = 0.79 > \phi_{\mathrm{MCT}}$ this was not possible. Below we present arguments that support the fact that the observed dynamical behaviour should remain unaffected inspite of not completely equilibrating the $\phi = 0.79$ sample.

1. To address whether under equilibration could have resulted in the absence of a two-slope behavior and a potentially larger $\xi_{\mathrm{d}}(\phi = 0.79) > \xi_{\mathrm{d}}(\phi = 0.76)$, we recalculate this quantity for $\phi = 0.76 \approx \phi_{\mathrm{MCT}}$ by considering only a small portion ($\approx 0.5\tau_\alpha$) of the total experimental data. By doing so we have effectively under equilibrated the sample. Supplementary Fig. 8 shows $\ln(\tau_{\mathrm{s}}(r)/\tau_{\mathrm{s}}^{\mathrm{bulk}})$ versus r for distinct pins. Although the statistics is poor due to the shorter data sets, $\xi_{\mathrm{d}}^{0.5\tau_\alpha}(\phi = 0.76) > \xi_{\mathrm{d}}(\phi = 0.79)$ and the non-monotonic evolution of ξ_{d} with ϕ persists. More importantly, we also observe the two-slope behavior, indicative of the multiple relaxation mechanisms anticipated by RFOT. This clearly implies that multiple-relaxation regimes cannot arise out of under equilibration and provides compelling evidence for a genuine change in relaxation process near ϕ_{MCT}. We emphasize here that despite being under equilibrated the $\phi = 0.79 > \phi_{\mathrm{MCT}}$ sample does not show the two-slope behaviour. Further, ξ_{stat} in both the self-induced pinning case as well as in the presence of pinned amorphous wall [2] grows systematically across ϕ_{MCT} and serves to compare and contrast with the striking non-monotonicity in ξ_{d}.

2. Supplementary Fig. 9 shows the self-intermediate scattering function $F_{\mathrm{s}}(q,t)$ at various wavevectors q. $F_{\mathrm{s}}(q,t)$ shows almost complete decay for $q = 0.5\sigma_{\mathrm{s}}$ (red solid triangles) which corresponds to the coarse-graining box size used in measuring ξ_{stat}, ξ_{d} and also Υ (see Supplementary Fig. 20). This indicates that the experimental duration is long enough for the sample to relax over the probe length scale determined by the coarse-graining box size. At smaller q's, we do not have sufficient long time data to show that $F_{\mathrm{s}}(q,t)$ eventually decays although the equilibration time (shown by the vertical gray region) is more than a decade larger than the time over which $F_{\mathrm{s}}(q,t)$ is determined.

3. Apart from the evidence for the existence of multiple relaxation regimes near ϕ_{MCT} being manifested by two-slopes (see Fig. 1d and Supplementary Fig. 8) numerous other quantities also show marked changes across ϕ_{MCT} [2]. The shapes of the top 10% most-mobile particle clusters change shape from string-like to compact across ϕ_{MCT}. The time scale over which these clusters are defined, t^* (the cage-breaking time), grows much more slowly with ϕ compared to τ_α [5]. Since the duration of the experiments is much larger than t^* at all ϕ including $\phi = 0.79$, we expect the observed trend in morphology of CRRs to depend only weakly on the equilibration time. Further,

the mobility transfer function, which is a measure of facilitated dynamics [6, 7] also shows a maximum in the vicinity of ϕ_{MCT} indicating that dynamical facilitation plays a secondary role in governing relaxation beyond ϕ_{MCT}.

4. Supplementary Fig. 10 shows the self-induced pins that are present at $3t^*$ (solid purple circles) and at $7t^*$ (hollow white circles). During the course of relaxation nearly 20% of the pins that were present at $3t^*$ did not survive until $7t^*$.

Supplementary Note 3 | Experimental realization of a pinned amorphous wall

The procedure for freezing particles in an amorphous wall configuration is described in detail in [2]. Briefly, a holographic optical tweezers setup is used to pin particles in the desired configuration. The set up consists of a linearly polarized constant power (800 mW) CW laser (Spectra-Physics, U.S.A., $\lambda = 1064$ nm) coupled via suitable optics to a spatial light modulator (Boulder Nonlinear Systems, Inc. U.S.A.). Particle coordinates within a strip of width $\sim 2\sigma_{\mathrm{L}}$ along the longer dimension of the field of view is obtained from a bright-field image. The hologram corresponding for creating optical traps at these coordinates is calculated using standard algorithms and fed into the spatial light modulator. This ensured that all particles corresponding to the frozen wall are trapped simultaneously. The particles making up the wall appear bright in the time-averaged images due to their negligible mobility. Importantly, the initial set of particle coordinates prior to optical trapping (color coded spheres) and the centres of pinned particles after optical trapping are separated by a distance smaller than the cage size, ensuring that the amorphous wall is indeed in an equilibrium configuration of the liquid (Supplementary Fig. 12).

Supplementary Note 4 | Calculation of instantaneous interface position from an amorphous wall and a self-induced pin

The amorphous wall is formed parallel to the x-axis of the field of view (Supplementary Fig. 12) [2] and is located at $z = 0$. Over a given τ_α, starting from each box located at $(x, z = 0)$ we scan along z and locate the box where the overlap dropped to $p_{\mathrm{c}}(\tau_\alpha) \leq 0.67$. A line through these boxes yields the instantaneous interface for a particular grid configuration. Next, the grids are displaced along various directions (see Section on Fuzzy-grid

method) and the instantaneous interface is calculated for each of the grid configurations. The spatial average of the interface positions from the nine realizations yielded the instantaneous interface for that particular τ_α and is represented by the height function $h(z,t)$. This procedure is followed for each τ_α and the interface dynamics are subsequently quantified.

Unlike the pinned amorphous wall case, where the interface is flat and parallel to the wall and hence easier to identify, the procedure for identifying interfaces for self-induced pins is more involved and is outlined below. Supplementary Fig. 13a shows an artificially generated interface (black line) separating regions of high configurational overlap (light green region) and low configurational overlap (yellow region). The self-induced pin is represented by the violet circle and is taken to be the origin. First, we scan along the $-z$-axis and locate the box where the overlap function was between $p_c(\tau_\alpha) \leq 0.67$ (yellow circle in Supplementary Fig. 13b). The coordinates of this box are $(0, -z_{\rm min})$. Next, for each box on the z-axis between 0 and $-z_{\rm min}$, we scan along the $+x$-axis and locate the boxes where the overlap fell between $p_c(\tau_\alpha) \leq 0.67$. A line through these boxes yields the interface (shown in red) in Supplementary Fig. 13b. Due to the jagged nature of the interface, certain regions of the interface are occluded from the x scan and this results in a discontinuous interface profile. To form the complete interface, we repeat scans in the orthogonal direction for all high overlap boxes located between 0 and $x_{\rm max}$. This once again resulted in a discontinuous interface profile, shown in yellow, in Supplementary Fig. 13c. This procedure is then repeated for the remaining quadrants. The interface position obtained from the individual scans are transformed to polar coordinates (r,θ) and then combined to obtain the complete interface profile (green interface in Supplementary Fig. 13d). If the interface profile is multivalued along a given scan direction, our algorithm identifies the coordinates that are nearest to the pin. This however, occurs only occasionally and does not influence our results. The interface profile is then interpolated in 1° increments.

The instantaneous interfaces are calculated for each displaced grid configuration (see section on Fuzzy-Grid method). The spatial average of all nine interface positions at each θ yields the instantaneous interface for the given τ_α and is represented by height function $h(r,\theta,t)$ from which the fluctuations are quantified. We then compute interfaces around different spatially well-separated pins for disorder averaging.

Supplementary Note 5 | Fourier decomposition of interface fluctuations

For the amorphous wall configuration, the interface fluctuations are calculated as $\Delta h(x,t) = h(x,t) - \langle h(x,t)\rangle_t$, where $\langle h(x,t)\rangle_t$ is the time-averaged baseline. Fourier decomposition of $\Delta h(x,t)$ yields the normal mode amplitudes $\langle |A(k)|^2\rangle$ (Supplementary Fig. 14) [4]. The interface length $l = 72\sigma_{\rm s}$, is decided by the size of the field of view and is a constant while performing the averaging.

In order to keep l nearly same while performing the averaging for the self-induced pin case, we follow the following protocol. For each instantaneous interface configuration, we find the average radius of the interface $r(t) = \langle h(r,\theta,t)\rangle_r$ and subsequently the interface length $l(t) = 2\pi r(t)$. The time-averaged radius of the interface for a given self-induced pin is $r_{\rm ave} = \langle r(t)\rangle_t$. We next identified those interface realizations which satisfied the condition $(2\pi r_{\rm ave} - \sigma_{\rm s}) \leq l(t) \leq (2\pi r_{\rm ave} + \sigma_{\rm s})$. Only these interfaces are considered for subsequent averaging. The time-averaged baseline for these interfaces is defined as $\langle h(r,\theta,t)\rangle_t$. Instantaneous interface fluctuations around the baseline are defined as $\Delta h = h(r,\theta,t) - \langle h(r,\theta,t)\rangle_t$. Subsequent Fourier decomposition and averaging yields the normal mode amplitudes $\langle |A(k)|^2\rangle$.

As mentioned earlier, the above procedure for identifying interfaces ignores overhangs (purple line in Supplementary Fig. 15a). Each individual mosaic typically contains more than one self-induced pin. We exploit this fact and perform an identical scan from a slightly displaced pin (Pin 2 in Supplementary Fig. 15b). As shown in the figure, during scans in the various quadrants starting from Pin 2, the interface overhang that was not captured earlier is now accessible. The surface tension estimated for five different pins within a single mosaic for $\phi = 0.79$ yield nearly identical values with $\Upsilon = 6.43 \pm 0.97 \times 10^{-14}$J/m. Ignoring overhangs leads to a very narrow spread in Υ and suggests that overhangs do not contribute significantly to the trends observed. More importantly, such overhangs are unlikely to pose a problem for the amorphous wall geometry and the nearly identical evolution of Υ with ϕ for both the wall and the self-induced pin further reinforces our claims.

Supplementary Note 6 | Identifying interfaces and calculation of Υ using $q_{\rm c}$

As mentioned in the main text the only difference between $q_{\rm c}(t)$ and $p_{\rm c}(t)$ is the manner in which the low overlap boxes are treated. In the former, a box that stay empty at $t = 0$

and $t = t$ is assigned an overlap value of 0, while in the latter the overlap takes the value 1. We naively expect that artificially assigning boxes with $q_c(t = \tau_\alpha)$ below a certain cut-off a value of 1 should yield results that are at least qualitatively similar to those obtained from $p_c(t = \tau_\alpha)$. To this end, boxes with $q_c(\tau_\alpha) \leq 0.15 \sim q_{\text{rand}}$ are assigned a value 1 and are also colored green in Supplementary Fig. 13 and are treated equivalent to high overlap boxes. Subsequently, we followed the procedure for identifying interfaces and calculating Υ laid out in the main text. Indeed, the behaviour of the interface width w and Υ for $\phi > 0.71$ using q_c yields results that are strikingly similar to those seen in Fig. 3 and Fig. 4 of the main text (Fig. S16-S19). At low ϕs, however, where particles in the liquid are highly mobile, the q_c analysis underestimates Υ.

Supplementary Note 7 | Surface tension measurements for $0.5\sigma_s$ boxes

To test the sensitivity of our surface tension (Υ) measurements on the coarse-graining grid size and also its dependence on the cut-offs we use for identifying high and low overlap regions, we recalculate Υ for a smaller box size of $0.5\sigma_s$ around the same self-induced pins analysed in the main text. The interfaces are determined for a low overlap criterion $0.2 \leq q_c \leq 0.084$ (q_{rand} for $0.5\sigma_s$). Computed quantities are represented in Supplementary Fig. 20. The trend in surface tension is similar to Fig.4d in the main text and a rapid growth across ϕ_{MCT} is also observed.

SUPPLEMENTARY REFERENCES

Berthier, L., and Kob, W., Static point-to-set correlations in glass-forming liquids. *Phys. Rev. E* **85**, 011102 (2012).

Nagamanasa, K. H., Gokhale, S., Sood, A. K., and Ganapathy, R., Direct measurements of growing amorphous order and non-monotonic dynamic correlations in a colloidal glass-former. *Nat. Phys.* **11**, 403-408 (2015).

Kob, W., Roldan-Vargas, S., and Berthier, L., Non-monotonic temperature evolution of dynamic correlations in glass-forming liquids. *Nat. Phys.* **8**, 164-167 (2012).

Gokhale, S., Nagamanasa, K.H., Santhosh V, Sood, A. K., and Ganapathy R., Directional grain growth from anisotropic kinetic roughening of grain boundaries in sheared colloidal crystals. *Proc. Natl Acad. Sci. USA* **109**, 20314-20319 (2012)

Starr, F. W., Douglas, J. F., and Sastry, S., The relationship of dynamical heterogeneity to the Adam-Gibbs and random first-order transition theories of glass formation. *J. Chem. Phys*, **138**, 12A541 (2013).

Gokhale, S., Sood, A. K., and Ganapathy, R., Deconstructing the glass transition through critical experiments on colloids. *Adv. Phys.* **65**, 363-453 (2016).

Elmatad, Y. S., and Keys, A. S., Manifestations of dynamical facilitation in glassy materials. *Phys. Rev. E* **85**, 061502 (2012).
