# Supplementary material for: Measurements of growing surface tension of amorphous–amorphous interfaces on approaching the colloidal glass transition
Source: Nat Commun. 2018 Jan 26;9:397. doi: 10.1038/s41467-018-02836-6 (PMC5786034; doi:10.1038/s41467-018-02836-6)
Supplement: Supplementary file 2 — Description of Additional Supplementary Files [file 41467_2018_2836_MOESM2_ESM.pdf]

Description of Additional Supplementary Files

File Name: Supplementary Movie1
Description:

Shows interface fluctuations in the wall geometry for ϕ=0.75 < ϕ_{MCT}. The background represents $p_c(t)$ over τ_α for one of the grid configurations. The color bar for the movie is same as in Fig. 3. The black line with red circles represents the instantaneous interface. Interfaces are more dynamic and have larger amplitude fluctuations in comparison to ϕ=0.79 > ϕ_{MCT} (see Movie S2). The interface, on an average, is also located closer to the wall in comparison to ϕ=0.79, consistent with the observed growth in the static length scale in Fig. 2c of the main text.

File Name: Supplementary Movie 2
Description:

Shows interface fluctuations in the wall geometry for ϕ=0.79 > ϕ_{MCT}. The background represents $p_c(t)$ over τ_α for one of the grid configurations. The color bar for the movie is same as in Fig. 3. The black line with red circles represents the instantaneous interface. The interface, on an average, is also located further away from the wall in comparison to ϕ=0.75 (Movie S1), consistent with the observed growth in the static length scale in Fig. 2c of the main text.

File Name: Supplementary Movie 3
Description:

Shows interface dynamics for self-induced pins for ϕ=0.75 < ϕ_{MCT}. The background image represents $p_c(t)$ over τ_α obtained after averaging over all the displaced grid configurations. The background image is an intensity averaged image. The interfaces shown, however, correspond averaging over their positions obtained for each grid configuration and hence need not necessarily have a one-to-one correspondence. The background serves only as a guide to the eye. The color bar for the movies is the same as Fig. 3. Red circles represent the self-induced pins. Different colors represent instantaneous interfaces corresponding to different pins.

File Name: Supplementary Movie 4
Description:

Shows interface dynamics for self-induced pins for ϕ=0.79 > ϕ_{MCT}. The background image represents $p_c(t)$ over τ_α obtained after averaging over all the displaced grid configurations. The background image is an intensity averaged image. The interfaces shown, however, correspond averaging over their positions obtained for each grid configuration and hence need not necessarily have a one-to-one correspondence. The background serves only as a guide to the eye. The color bar for the movies is the same as Fig. 3. Red circles represent the self-induced pins. Different colors represent instantaneous interfaces corresponding to different pins. For $\phi \approx> \phi_{MCT}$, distinct high persistence regions have fairly well-defined boundaries between them. The high persistence regions also appear to tile space beyond ϕ_{MCT}.
